# Blocking Zika virus vertical transmission

**DOI:** 10.1038/s41598-018-19526-4

**Published:** 2018-01-19

**Authors:** Pinar Mesci, Angela Macia, Spencer M. Moore, Sergey A. Shiryaev, Antonella Pinto, Chun-Teng Huang, Leon Tejwani, Isabella R. Fernandes, Nicole A. Suarez, Matthew J. Kolar, Sandro Montefusco, Scott C. Rosenberg, Roberto H. Herai, Fernanda R. Cugola, Fabiele B. Russo, Nicholas Sheets, Alan Saghatelian, Sujan Shresta, Jeremiah D. Momper, Jair L. Siqueira-Neto, Kevin D. Corbett, Patricia C. B. Beltrão-Braga, Alexey V. Terskikh, Alysson R. Muotri

**Affiliations:** 1 University of California San Diego, School of Medicine, Department of Pediatrics/Rady Children’s Hospital San Diego, Department of Cellular & Molecular Medicine, Stem Cell Program, La Jolla, CA 92037–0695 USA; 2 Sanford Burnham Prebys Medical Discovery Institute, 10901N. Torrey Pines Rd., La Jolla, CA 92037 USA; 3 Salk Institute for Biological Studies, Clayton Foundation Laboratories for Peptide Biology, Helmsley Center for Genomic Medicine, La Jolla, California USA; 4 University of California San Diego, Skaggs School of Pharmacy and Pharmaceutical Sciences, Center for Discovery and Innovation in Parasitic Diseases, 9500 Gilman Dr., La Jolla, CA 92093, MC 0755 USA; 5 Ludwig Institute for Cancer Research, San Diego Branch, 9500 Gilman Dr., La Jolla, CA 92093, MC 2385 USA; 6 University of California San Diego, Department of Cellular and Molecular Medicine, 9500 Gilman Dr., La Jolla, CA 92093, MC 2385 USA; 7 Graduate Program in Health Sciences, School of Medicine, Pontifícia Universidade Católica do Paraná (PUCPR), Curitiba, Paraná, Brazil; 8 University of São Paulo, Institute of Biomedical Science, Department of Microbiology, Laboratory of Stem Cell and Disease Modeling, São Paulo, SP 05508–000 Brazil; 9 University of São Paulo, School of Arts Sciences and Humanities, Department of Obstetrics, São Paulo, SP 03828–000 Brazil; 10 University of São Paulo, School of Medicine, Center for Cellular and Molecular Therapy (NETCEM), São Paulo, SP 01246–903 Brazil; 11 Division of Inflammation Biology, La Jolla Institute for Allergy & Immunology, La Jolla, CA 92037 USA; 12 Skaggs School of Pharmacy and Pharmaceutical Sciences, University of California, San Diego, La Jolla, CA 92093 USA

## Abstract

The outbreak of the Zika virus (ZIKV) has been associated with increased incidence of congenital malformations. Although recent efforts have focused on vaccine development, treatments for infected individuals are needed urgently. Sofosbuvir (SOF), an FDA-approved nucleotide analog inhibitor of the Hepatitis C (HCV) RNA-dependent RNA polymerase (RdRp) was recently shown to be protective against ZIKV both *in vitro* and *in vivo*. Here, we show that SOF protected human neural progenitor cells (NPC) and 3D neurospheres from ZIKV infection-mediated cell death and importantly restored the antiviral immune response in NPCs. *In vivo*, SOF treatment post-infection (p.i.) decreased viral burden in an immunodeficient mouse model. Finally, we show for the first time that acute SOF treatment of pregnant dams p.i. was well-tolerated and prevented vertical transmission of the virus to the fetus. Taken together, our data confirmed SOF-mediated sparing of human neural cell types from ZIKV-mediated cell death *in vitro* and reduced viral burden *in vivo* in animal models of chronic infection and vertical transmission, strengthening the growing body of evidence for SOF anti-ZIKV activity.

## Introduction

The outbreak of the Zika virus (ZIKV) has been associated with an increase in newborns with congenital malformations in Brazil in the past two years^1,2^. A new report describing 13 infants born in Brazil between 2015 and 2016 showed that although they did not have microcephaly at birth, they subsequently had head growth deceleration to the point of microcephaly concomitant with significant neurologic abnormalities^3^.

Given the growing threat of ZIKV spread, some researchers have focused on prophylactic measures by screening FDA- approved drugs to repurpose for ZIKV^4–6^, while still other researchers worldwide have focused on vaccine development. Accordingly, a DNA vaccine to protect against ZIKV was recently proposed, with promising results *in vivo*^7,8^. While immunization initiatives are important, there is a need to develop clinical strategies to treat ZIKV-infected individuals, including pregnant women for whom prevention of infection is no longer an option. Indeed ZIKV infection during the first trimester confers the greatest risk of congenital microcephaly, thus highlighting the urgent need for treatment of infected mothers^9^.

To provide a potential treatment against the detrimental effects of ZIKV infection we focused on testing the antiviral Sofosbuvir (SOF), a clinically-approved RNA-dependent RNA polymerase (RdRp) inhibitor, both *in vitro* with ZIKV-infected human neural progenitor cells (NPCs) and *in vivo* with mouse models. Recently, converging evidence that SOF is protective against ZIKV infection in different cell types was reported, although the precise gene expression-level events in target cells have not been described^10–13^. SOF is a nucleotide analog pro-drug used in combination with other drugs for the treatment and cure of chronic infection with hepatitis C virus (HCV), a member of *Flaviviridae* family^14–18^. Upon intracellular conversion to its active triphosphate, SOF acts on HCV by inhibiting its RNA polymerase, inhibiting replication^14,15^. We found that the RNA polymerases of HCV and ZIKV showed strong similarities in their active sites, and thus confirmed that SOF could also act on ZIKV-infected cells. Our data corroborated the findings of other groups^19,20^ and expand on these findings by showing that SOF treatment rescued apoptotic human NPCs infected with multiple ZIKV strains, both in monolayer and 3D culture. In addition, SOF also restored antiviral response gene expression in human NPCs. Furthermore; we found that SOF inhibited ZIKV more efficiently in NPCs than in Vero cells. SOF was previously shown to be protective in interferon-response compromised mouse models and in neonatal Swiss mice^12,13^. To extend these findings to another animal model, we chose a mouse compromised in adaptive immunity to test SOF. We found that while the placebo group had significantly increased viral load, SOF-treated mice had reduced serum ZIKV. Finally, we tested the efficacy of SOF in blocking viral vertical transmission in the ZIKV-permissive SJL strain^21^. Importantly, viral burden was reduced in SOF-treated dam serum during pregnancy. Fetuses of SOF-treated dams did not show detectable ZIKV amplification, suggesting that SOF was (1) well-tolerated by ZIKV-infected SJL dams and, most importantly, (2) was able to arrest ZIKV replication *in vivo* in pregnant SJL females abolishing vertical transmission. The results presented here demonstrate that repurposing the FDA-approved antiviral SOF has efficacy against ZIKV *in vitro* and *in vivo* and warrants further molecular and translational investigation.

## Results

### RdRp domain is highly conserved between Hepatitis C and Zika virus

**Figure 1:**
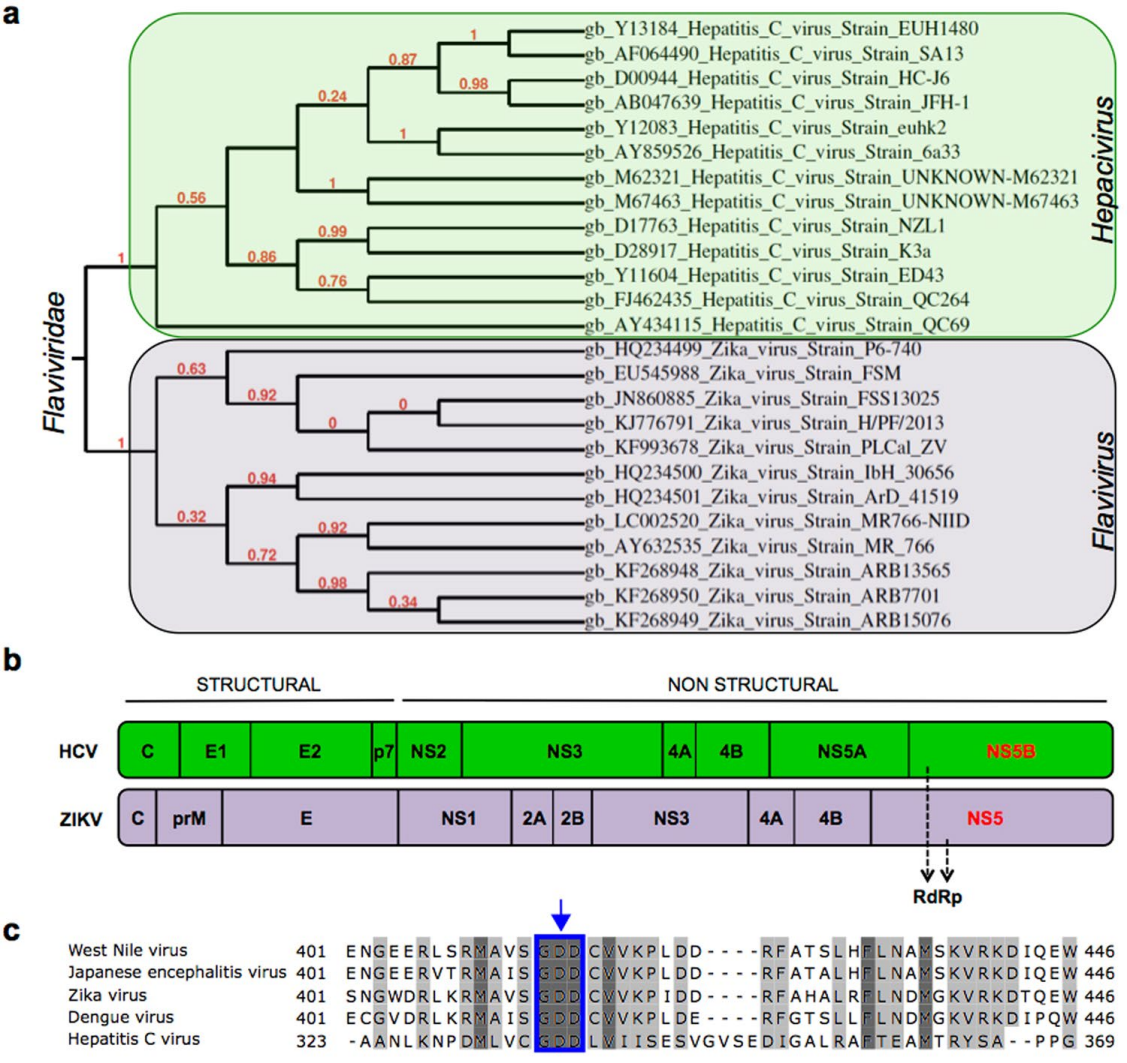
The RdRp domain is conserved between HCV and ZIKV. (**a**) Phylogenetic dendrogram showing HCV and ZIKV belonging to *Hepacivirus* and *Flavivirus* genera, respectively, within the *Flaviviridae* family. Phylogenetic tree of RNA polymerase domain of HCV and ZIKV strains was based on Maximum-likelihood approach. This tree is intended to highlight the degree of similarity (branch length) of the RNA polymerase from different viruses. The longer the branch is in the horizontal dimension, the larger the amount of change. The units of branch length are amino acid substitutions per site. (**b**) Schematic of HCV and ZIKV protein structures. Both viruses have their RNA-dependent polymerases (RdRp) within the NS5B and NS5 domains, respectively. (**c**) Capsid protein; prM: precursor of Membrane protein; E: Envelope protein; p7: Viroporin, NS: Non-structural proteins. (**c**) RNA polymerase amino acid sequence conservation and DPP domain between different members of *Flaviviradae* family including HCV, West Nile virus, Japanese encephalitis, Dengue virus and ZIKV. Highlighted sequences correspond to amino acid sequence conservation between strains. Light gray corresponds to amino acid conservation in 4 strains, and dark gray correspond to amino acid conservation in 5 strains (all represented strains). The blue box indicated by the arrow corresponds to the GDD amino acid domain that is conserved in all 5 represented virus strains (highlighted as dark gray).

The *Flaviviridae* viral family includes both HCV and ZIKV^22^. Both HCV and ZIKV RdRp-encoding genes lie within NS5B and NS5 domains of the viral genome, respectively. These domains are highly conserved, especially in the active site residues, among *Flaviviridae* members^8,22^ (Fig. 1a–c). The NS5B C-terminal GDD motif catalyzes nucleotidyl transfer (Fig. 1c) and is highly conserved in *Flaviviridae* family (Fig. 1a–c; Supplementary Fig. 1A–B). The high sequence homology among the *Flaviviridae* RdRps suggests the possibility for a common target and mechanism of action. SOF is a 2′-modified nucleotide analog, and upon intracellular phosphorylation from its prodrug state, inhibits catalysis by incorporating into the growing RNA chain and terminating synthesis^17^. Since SOF-triphosphate inhibits the HCV RdRp^14,23^ and given the conserved structural similarities of the RdRp domain within the *Flaviviridae* family, we confirmed that SOF could also inhibit ZIKV replication by targeting RdRp (Fig. 1b–c). Recently, the crystal structure of the ZIKV NS5 protein was released (PDB ID: 5TFR; Longenecker, K.L., Upadhyay, A.K., Cyr, M., http://www.rcsb.org/pdb). We overlaid the ZIKV NS5 (containing the RdRp domain) structural model onto a recent structure of HCV NS5B bound to dsRNA and SOF (PDB ID 4WTG^23^) (Supplementary Fig. 1C, Supplementary Video 1). This comparison shows that the active sites of the two polymerases are indeed similar, with all residues contacting SOF conserved between HCV NS5B and ZIKV NS5. The close structural similarity suggests that SOF could interact with ZIKV NS5 and could inhibit its RNA polymerase activity (Supplementary Fig. 1C, Supplementary Video 1).

### Sofosbuvir inhibits ZIKV replication in human iPSC-derived NPCs

**Figure 2:**
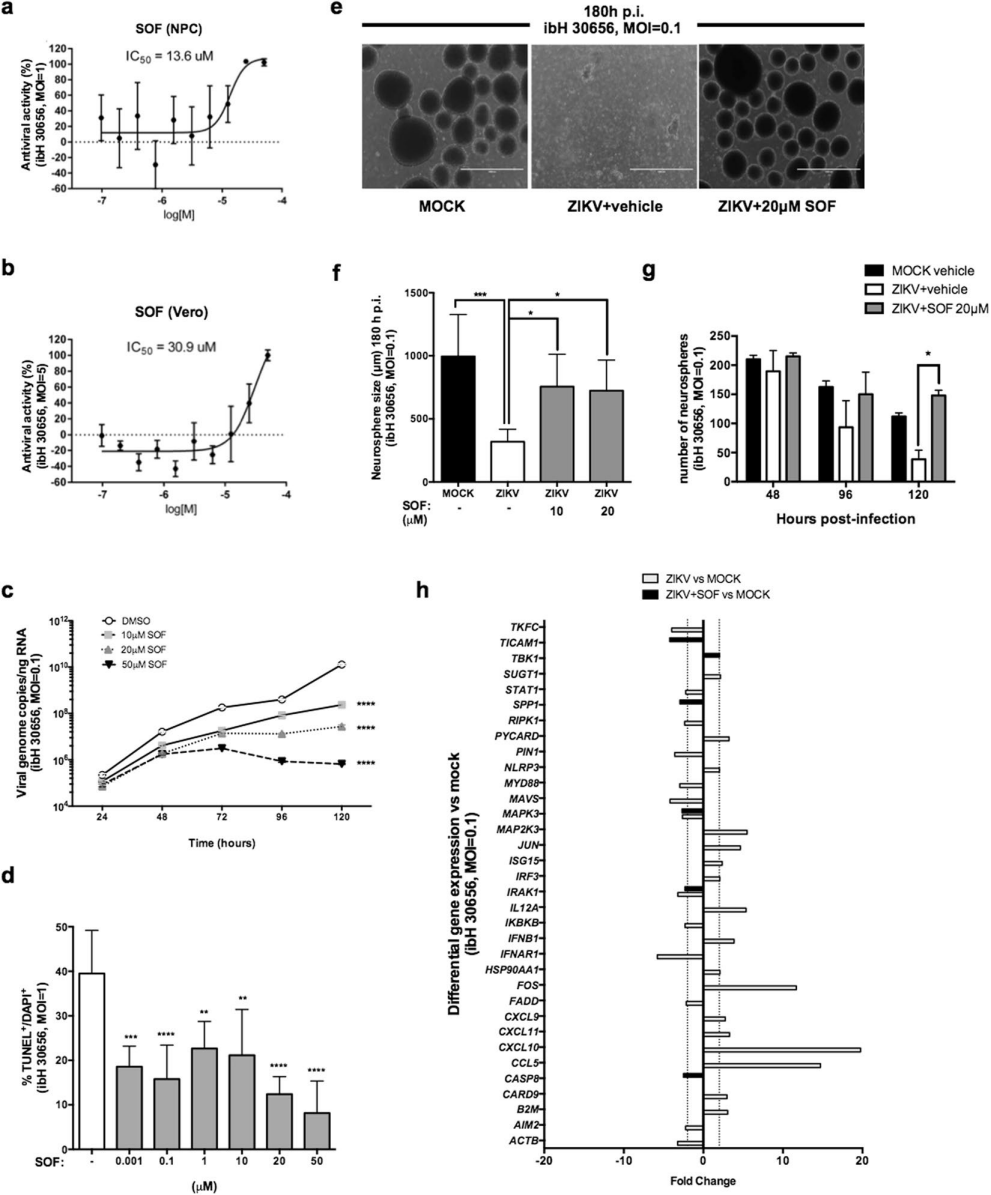
SOF blocks ZIKV replication *in vitro*. (**a**,**b**) Dose-response curves of SOF against ZIKV in NPCs and in Vero cells, respectively. SOF was tested in 10 points dose-response with two-fold dilution starting at 50 μM, in triplicates. The graphs show the antiviral activity (%) based on reduction of the cytopathic effect by quantification of the cytopathic effects of ZIKV ± SEM on NPCs (**a**) and Vero cells (**b**,**c**). Multi-step growth curve of vehicle or SOF-treated ZIKV-infected (ibH 30656, MOI = 0.1) NPCs at different concentrations over time as measured by viral genome copies of RNA using qRT-PCR. Data represents means ± SD (n = 3 replicates); two-way ANOVA, followed by Tukey’s multiple comparison tests. ****P < 0.0001 compared to DMSO (vehicle) treated NPCs at 120 hours p.i. Note that SD cannot be seen in the log scale. (**d**) The percentages of apoptotic cells were calculated, averaged, and graphed accordingly (ibH 30656, MOI = 0.1, 96 hours p.i.) in the presence of SOF at different doses (1 nM, 100 nM, 1 μM, 10 μM, 20 μM and 50 μM). One-way ANOVA tests with Tukey multiple comparisons were performed to compare to ZIKV-infected different groups. The presented values are means of TUNEL^+^/DAPI^+^ percentage ± SD (n = 6 images per condition), **P < 0.01, ***P < 0.001, ****P < 0.0001 TUNEL^+^/DAPI^+^ percentage decreases with different doses of SOF. (**e**) Representative images of mock-infected human neurospheres (left panel), ZIKV-infected vehicle-treated neurospheres (middle panel) and ZIKV-infected SOF-treated (20 μM) (right panel) neurospheres image (ibH30656, MOI = 0.1, 180 hours p.i.), scale bar: 500 μm. (**f**) Quantification of neurosphere diameter (μm) at 180 hours p.i. One-way ANOVA tests with Tukey multiple comparison were performed to compare different groups. The bars represent the number of spheres counted averaged and plotted for each condition ± SD (n = 6 images captured per condition), *P < 0.05, ***P < 0.001 (n ≥ 100 neurospheres counted per condition). (**g**) Neurosphere amount was counted over time after infection with ZIKV (ibH30656, MOI = 0.1 at 48, 96 and 120 hours p.i.) treated with vehicle or with SOF (20 μM). Bars represent the number of neurospheres ± SD at a given time (48, 96 and 120 hours p.i.). Two-way ANOVA tests with Tukey multiple comparison were performed to compare different groups, *P < 0.05 (n = 2 independent experiments). (**h**) Human antiviral response expression signature in human NPCs in monolayer at 120 hours p.i.: mock, ZIKV-infected (ibH30656, MOI 0.1) vehicle or SOF-treated (50 μM) (threshold = two-fold). Bars represent fold changes detected by qPCR from 96 different genes tested, between ZIKV-infected compared to mock-infected NPCs (in white) and between ZIKV + SOF-treated compared to mock-infected NPCs (in black). Note that there are fewer dysregulated genes between ZIKV-infected compared to mock NPCs upon SOF treatment (less black bars) than the ZIKV-infected untreated ones.

Previous work has demonstrated that ZIKV infection slows growth of human neural progenitor cells (NPCs) and neurospheres *in vitro*^21,24–26^ and adversely affects mouse neural progenitors and radial glia^19,27^. To test if SOF arrests ZIKV replication, human induced pluripotent stem cell (hiPSC)-derived NPCs were chosen as the cellular model since they mimic the proliferative growth restrictions leading to microcephaly^21^. SOF blocked ZIKV activity with IC_50_s of 13.6 μM and 30.9 μM in NPCs and Vero cells (used as a cell-based infection system), respectively (Fig. 2a,b). The antiviral effect of varying concentrations of SOF was further assessed in multi-step growth curves on ZIKV-infected NPCs by qRT-PCR of viral genome copies (Fig. 2C, Supplementary Table S1). Next, we evaluated whether SOF treatment could rescue the apoptotic phenotype of ZIKV-infected NPCs. NPCs were infected with ZIKV MOI 0.1 or 1, 24 hours after plating and maintained 96 hours in culture. In mock-infected conditions, around 15% of cells were found to be apoptotic (Fig. 2d; Supplementary Fig. 2A–C). Upon ZIKV infection with an MOI 0.1 or 1, the percentage of TUNEL^+^ NPCs significantly increased to 25–40% (Fig. 2d), similarly to previous observations^21^. SOF treatment in the mock-infection had no effect on apoptosis (Supplementary Fig. 2B). SOF decreased significantly the percentage of TUNEL^+^ NPCs at different doses tested (Fig. 2d). In addition, SOF significantly decreased the expression of the early apoptotic marker, cleaved caspase 3 (CC3), in ZIKV-infected hiPSC-derived NPCs and hiPSC-derived cortical neurons, using a different viral strain with higher MOI (Brazil-ZKV 2015, MOI 10) (Supplementary Fig. 2D–F).

We next assessed the ability of NPCs to form tridimensional neurospheres upon ZIKV infection. As previously shown^21^, ZIKV-infected vehicle-treated neurospheres showed reduced size (diameter) and viability (number of surviving neurospheres) over time, but SOF treatment at different doses successfully rescued these phenotypes (Fig. 2e–g; Supplementary Fig. 3A–D). To measure SOF efficacy in rescuing molecular phenotypes, we measured the antiviral response gene expression signature in human NPCs in monolayer at 120 hours post-infection (p.i.) (Fig. 2h; Supplementary Fig. 3e). We found several dysregulated genes upon ZIKV infection: including but not limited to increased expression of *CCL5*, *CXCL9*, *CXCL10*, *CXCL11*, *MAP2K3*, *IFNB1*, *ISG15*, *NLRP3* and decreased expression of *IKBKB*, *IRAK1*, *PIN1*, *MYD88;* this pattern of gene dysregulation in response to ZIKV infection serves as a biomarker, reversal of which would be a desirable attribute of efficacious therapy. SOF treatment reversed most of the dysregulation of antiviral response genes tested, showing a similar gene expression pattern between SOF-treated ZIKV-infected NPCs and mock-infected NPCs (Fig. 2h; Supplementary Fig. 3E). In conclusion, our *in vitro* studies suggest that SOF blocks replication of different ZIKV strains, supporting our *in silico* predictions and confirming previous publications^10,12,28–30^.

### SOF rescues Zika viral burden in mice

**Figure 3:**
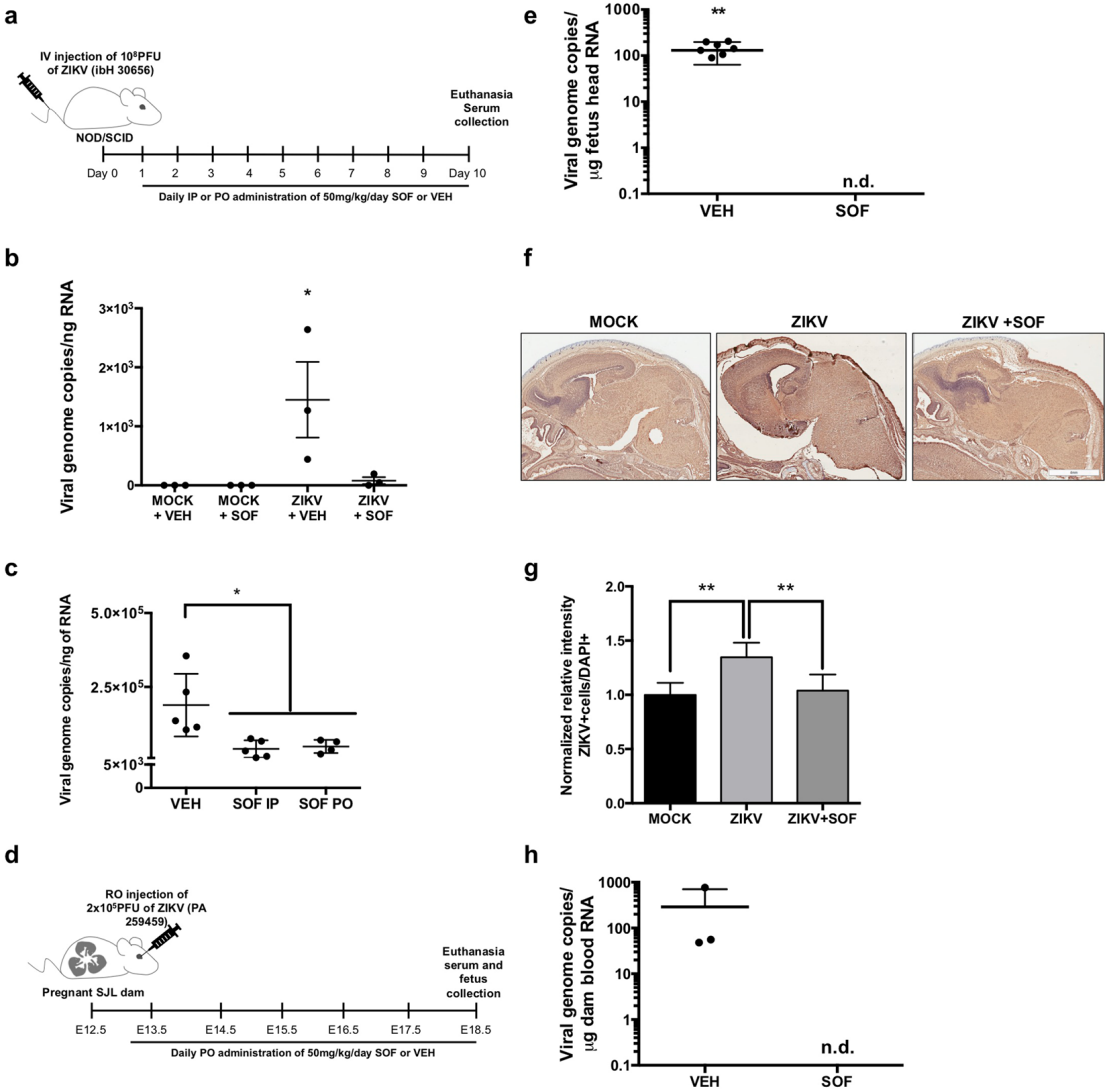
SOF blocks ZIKV replication *in vivo*. (**a**) Schematic of the experimental design for mice infection: 8 weeks-old NOD/SCID female mice were intra-venously injected with 10^8^ PFU of ZIKV (ibH 30656). One day p.i. mice were randomized to receive either vehicle or SOF at 50 mg/kg/day IP or PO. (**b**) RNA viral load was measured in the serum of infected mice by qRT-PCR. One-way ANOVA tests with Tukey multiple comparison were performed to compare to MOCK-vehicle (VEH) group, *P < 0.05 (n = 3 technical replicates, n = 3 mice per group). Each dot represents the average of n = 3 technical replicates per mouse ± SEM. (**c)** RNA viral load was measured in the serum of infected mice by qRT-PCR. Each dot represents a mouse: vehicle n = 5 mice, SOF IP n = 5 mice, SOF PO n = 5 and monitored for 10 days before euthanasia and blood collection. One-way ANOVA tests with Tukey multiple comparison were performed to compare to vehicle group, *P < 0.05. Bars represent ± SD. (**d**) Schematic of the experimental design for infection of SJL pregnant mice. SJL dams were ZIKV-infected (PA 259459) with 2 × 10^5^ PFU at E12.5. At E13.5, they were randomly assigned to receive vehicle or SOF (50 mg/kg/day) PO. At E18.5, mice were euthanized for blood and fetus collection. (**e**) RNA viral load was measured in the fetus heads mice by qRT-PCR. Each dot represents a fetus head (vehicle-treated n = 8 pooled from 3 independent litters, 1 did not amplify and SOF-treated, n = 6 fetus heads from 3 independent litters). Student’s t-test was performed to compare the two groups; Bars represent the average viral load ± SD (**P < 0.01), n. d. = not detected. (**f**) Immunohistochemistry against Flavivirus Group Antigen (brown) and counterstained in Mayer’s hematoxylin (blue) in brain cross-sections of E18.5 fetuses from mock-infected, ZIKV-infected vehicle-treated, or ZIKV-infected, SOF-treated dams. Scale bars 4 mm. (**g**) All data are represented as mean ± SD (n = 6 per condition: 3 embryos, 2 slides each embryo per condition) and were analyzed with one-way ANOVA (Turkey’s Multiple Comparison post test). **P < 0.01. (**h**) RNA viral load was measured in the dam serum by qRT-PCR. Each dot represents one mouse ± SD (vehicle-treated n = 3 and SOF-treated, n = 3). n. d. = not detected. Student’s t-test was performed to compare the two groups (*P* = 0.2902).

Next, we assessed the efficacy of SOF *in vivo*. While this manuscript was under preparation, SOF was shown to protect against ZIKV in immunocompetent mice treated with an anti-IFNAR1 blocking antibody and in neonatal Swiss mice^12,13^, However, the first model has major limitations, as 20% of untreated ZIKV-infected mice survived, questioning the utility and efficiency of interferon (IFN)-response depleted mice to test drugs against ZIKV infections. And the second model only used neonatal pups for ZIKV infections. Thus, we chose the NOD/SCID adult immunodeficient mouse as a model for ZIKV infection (Fig. 3a). NOD/SCID mice can control the initial viral burden through an intact interferon response. However, this mouse model lacks functional T or B cells and has defective natural killer cells, a class of cytotoxic innate lymphocytes that rapidly respond to virally infected cells in the absence of antibodies, mimicking better the human condition^31,32^. Another advantage of the NOD/SCID model is the lack of confounding antibody response in testing prospective antiviral agents. A previous study compared survival after ZIKV infection in two mouse models, IFN-α/β and IFN-γ receptor knockout AG129 and SCID^32^, and found that AG129 mice survived 18.5 days while SCID mice survived 40 days following an identical infection of 2 × 10^3^ PFU/ml. These data suggest that animal survival depends on the viral load and adaptive immunity and not solely IFN response^32^. In our study, NOD/SCID mice were infected with 10^8^ PFU ZIKV intravenously (IV), and started to lose weight around day 11 with mean survival of 21± 3.36 days (Supplementary Fig. 4A–C). ZIKV infected animals progressively developed severe disease requiring euthanasia (weight loss, hunched back, hindlimb paralysis). In a subsequent experiment mice were randomized to receive vehicle or SOF 50 mg/kg/day intraperitoneally (IP) beginning at day 1 p.i., for 10 days, to study antiviral activity at peak viremia^33–35^ (Fig. 3a). The dosage of SOF (50 mg/kg/day) used in our *in vivo* experiments was determined after calculating the animal equivalent dose (AED) from the recommended 400 mg/day dose for chronic HCV infection in humans. This calculation takes into account the lower metabolic rate in larger animals, requiring a smaller drug per body weight dose among other criterion^36^. SOF treatment was well tolerated in mice: no marked changes in body weight (Supplementary Fig. 4D), fur, consistency of the stool or behavior (data not shown) during the treatment similar to previous work^18^. All mice from the ZIKV-infected vehicle-treated group had increased serum viral load, measured by plaque assays. Serum from ZIKV-infected SOF-treated mice failed to form plaques (Supplementary Fig. 4E,F). Consistently, qRT-PCR of viral RNA extracted from the serum of ZIKV-infected vehicle-treated mice revealed high levels of viral RNA. In contrast, SOF-treated ZIKV-infected mice had significantly less ZIKV RNA (Fig. 3b, Supplementary Table S2).

SOF is indicated for oral (PO) administration in humans. In healthy subjects administered the clinical SOF dose of 400 mg PO daily, average steady state concentrations of the predominant circulating metabolite GS-331007 were 463 ng/mL^37^. Hence, we considered whether SOF exposure depends on route of administration (PO versus IP). We delivered 50 mg/kg/day SOF to naïve mice daily for 4 days by IP or PO (Supplementary Fig. 4G) After 4 days, sera were extracted and subjected to liquid chromatography/mass spectrometry analysis, demonstrating average minimum serum concentrations (C_min_) of the predominant circulating metabolite GS-331007 of 608 and 588 ng/mL in the IP and PO-treated groups, respectively, with no significant differences between groups, suggesting that SOF has antiviral activity against ZIKV at concentrations that have been shown to be safe in humans. Indeed, when we repeated the initial experiment with NOD/SCID mice infected with ZIKV to compare the efficacy of PO versus IP, both delivery routes significantly decreased viremia after 10 days in a similar fashion (Fig. 3c).

Finally, as the abolition of vertical transmission is of critical interest in the context of congenital ZIKV infection, we tested the efficacy of SOF to block ZIKV vertical transmission in the ZIKV-permissive SJL strain^21^. Pregnant females were infected with 2 × 10^5^ PFU (Asian ZIKV strain PA 259459) at embryonic day (E) 12.5 as previously described^38^. Dose and timing of the viral inoculum were selected based on our previous publication and caused a similarly robust ZIKV infection of SJL dams, corroborating previous work^38^. The viral inoculation dose was selected as comparable to vector-borne inoculation^39^ (Fig. 3d; Supplementary Fig. 4A). The mice were randomized to receive 50 mg/kg/day of SOF or vehicle PO from E13.5–E18.5, when they were euthanized and the fetuses extracted (Fig. 3d). The viral RNA was detected in all vehicle-treated dam sera at E18.5, and in heads of their respective fetuses (Fig. 3e). Immunohistochemistry against Flavivirus Group Antigen in brain cross-sections of E18.5 embryos coming from SOF-treated dams showed a reduction of ZIKV compared to fetuses from VEH-treated dams (Fig. 3f,g). In addition, no obvious congenital malformations or weight loss were detected between fetal groups at this stage when infected with ZIKV at 2 × 10^5^ PFU (Supplementary Fig. 4H). The viral burden was significantly reduced in SOF-treated dam serum at E18.5 (Fig. 3h). Fetuses of SOF-treated dams did not show any detectable ZIKV amplification, suggesting that SOF was (1) well-tolerated by ZIKV-infected SJL dams and, most importantly, (2) was able to arrest ZIKV replication *in vivo* in pregnant SJL females abolishing vertical transmission.

## Discussion

Here we obtained similar results regarding the reduction in ZIKV infection-induced cell death in a 2D model, and a rescue of ZIKV-mediated decreased neurosphere size in 3D cultures with SOF treatment, adding to the mounting evidence that SOF can act against ZIKV^10,12,28–30,40^. In addition, we show that treatment with SOF further restored the antiviral response gene expression signature of ZIKV-infected NPCs. We also tested SOF in an *in vivo* immunodeficient mouse model. SOF treatment reduced serum ZIKV burden in all treated mice. Importantly, we show here for the first time that SOF was also well tolerated in pregnant dams and abolished vertical transmission of ZIKV to the fetuses.

The prevailing ZIKV model describes a causal relationship between maternal infection, neonatal microcephaly and motor defects manifesting several months after birth^41^, thus implicating vertical transmission^21,27^. Therefore, effective treatment must focus on reducing viral replication in pregnant women and thus the risk of vertical transmission to the fetus. However, it is still unclear how vertical transmission occurs *in vivo*. Recently, we generated hiPSC-derived microglia and NPCs to mimic and study the neuro-immune interactions during embryogenesis^29^. Microglia, the macrophages of the central nervous system (CNS), are generated in the yolk sac and invade the brain during embryogenesis to become resident macrophages^42^. Given the timing of their invasion, microglia could spread the virus in the developing brain during its CNS infiltration acting as a Trojan horse^29^. We found that microglial cells could indeed transmit ZIKV to NPCs *in vitro* and importantly, when microglia-NPC co-cultures were treated with SOF, ZIKV infection of NPCs was limited^29^. Although out of scope of the present study, one limitation of our work is the lack of congenital disease, showing that SOF treatment can prevent birth defects in the newborn pups. To this end, a recent study treated neonatally ZIKV-infected pups with SOF and showed that short- and long-term sequelae could be prevented^13^, thus supporting our hypothesis regarding the benefits of SOF treatment of ZIKV- infected pregnant women. Future studies should focus on the treatment of congenital disease using SOF and analysis of other tissues such as placenta, spleen, thymus etc. Taken together with our current study, there is strong converging evidence that SOF is a reasonable option to block vertical transmission, possibly acting by limiting the infection of microglia thus restricting viral spread to the fetal brain. SOF is labeled as FDA pregnancy category B, indicating that while no controlled studies have been conducted in pregnant women receiving the drug, animal studies *have not demonstrated risks to the fetus*^16,18^. Indeed, a clinical trial has recently commenced recruiting pregnant women with chronic HCV (NCT02683005) to study the effects of SOF during pregnancy. Comparable nucleotide/nucleoside analog antivirals such as acyclovir (herpes simplex virus) and the HIV anti-retrovirals are routinely used during pregnancy to prevent perinatal transmission and have not shown elevated risks of birth defects^43,44^. The placental transfer and tissue distribution of SOF and related metabolites was previously evaluated using quantitative whole body autoradiography in pregnant female rats administered a single 20 mg/kg oral dose of ^14^C-sofosbuvir^45^. Importantly, SOF-derived radioactivity was detected in fetal brain with exposures that were 5-fold higher than those observed in pregnant dams at 24 hours post-dose. This finding indicates that SOF may have sufficient brain penetration to inhibit viral replication in the developing fetal brain while also achieving systemic exposure necessary to block replication at other sites. No deleterious SOF effects on fetal development have been observed at the highest doses tested in gestating rats and rabbits (Gilead Sciences). Interestingly, a study showed that SOF delivered at 44 mg/kg/d or 440 mg/kg/d for 14 days did not cause liver toxicity in the chimeric TK-NOG mice with humanized livers^18^. Pregnancy category B states a treatment should be used during pregnancy only if the potential for benefit justifies the potential risk to the fetus^46^. However, in pre-clinical reproduction toxicity studies, SOF had no effects on embryo-fetal and pre-and post-natal development at exposures at least 10-fold greater than the mean clinical exposure at the approved SOF dose of 400 mg (Sofosbuvir For Treatment of Chronic Hepatitis C Infection Antiviral Drugs Advisory Committee Meeting Briefing Document, Gilead Sciences, 2013). Moreover, SOF has been shown to be brain and placenta-penetrant^47^. In line with this, our data indicate that SOF abolishes vertical ZIKV transmission in pregnant immunocompetent mice. Although the animal dosages (50 mg/kg) used experimentally exceed the human dose (400 mg) by weight, species scaling by body surface area corresponds to a scaled human equivalent dose (HED) of 4 mg/kg, less than the human SOF dose for a 70 kg individual^36^. Therefore, our results suggest that SOF used as a treatment for ZIKV could be prescribed at the same dosage as it is used for HCV patients.

Finally, in addition to the risks in pregnant women, in adults, a high incidence of Guillain-Barré syndrome has been linked to ZIKV infection^48^, as well as other ZIKV-infection sequelae, which include meningoencephalitis^49^, uveitis^50^, and acute myelitis^51^. It is possible that other clinical manifestations of ZIKV infection will surface in the near future. Given our results, treatment with SOF could also arrest or help to prevent the development of other clinical manifestations observed in adults.

Research in the use of nucleotide/nucleoside analog inhibitors to combat ZIKV infection has accelerated in the last year, with demonstrated antiviral activity among 2′-*C*-methylated nucleosides in cultured Vero cells and in a mouse model^32,52^. In this study, we took advantage of the similarities between the RdRp domain of HCV and ZIKV and hypothesized that SOF; an FDA-approved drug indicated for treatment of chronic HCV infection, could also arrest ZIKV replication. Our work and others’ recently published studies, have shown SOF to be protective against different strains of ZIKV including the strain isolated from a microcephalic case in Brazil, used in the present study along with others. However, both our work and that of others have found a higher IC_50_ of SOF against ZIKV when compared to HCV. Interestingly, we found the IC_50_ of SOF against ZIKV in human NPCs to be lower than in Vero cells, suggesting greater potency in this cell type. Nonetheless, our calculated IC_50_ values against SOF suggest reduced potency against ZIKV versus HCV. This is perhaps not surprising given that SOF was initially designed for chronic HCV infection targeting the liver - a different virus and different cell type versus our models, thus highlighting potential cell-type specific differences in drug metabolism^37^ (Sovaldi ® Full Prescribing Information, Gilead Sciences, Inc, 2013). Other SOF analogs tested both *in vitro* and *in vivo* have shown greater potency than SOF^32,52,53^. Future research should focus on developing ZIKV-specific SOF analogs. Nonetheless, currently SOF remains the only FDA-approved drug readily available and *is still protective against ZIKV* as we and other groups have shown^10–13,52,53^. SOF regimen for HCV infection is very costly in the United States, though it is less expensive in low-income countries such as Brazil, and a generic version could address the imminent public concern of the Zika outbreak. However, although the cost of SOF regimen is lower in developing countries, it still remains out of reach for many individuals in low-income areas to treat HCV-infected patients, making it even more challenging to treat ZIKV-infected individuals. Thus, there is an urgent need to make SOF less costly worldwide, which could increase assistance to HCV- and ZIKV-infected patients. Thus, our results warrant further study of the immediate application of SOF treatment to ZIKV-infected adults since no other treatments are currently available.

In conclusion, our data illustrate the advantage of immediate translational potential through repurposing the nucleotide analog SOF that is already in wide clinical use for chronic HCV infection. In the wake of limited outcome data available for long-term effects of congenital ZIKV and no therapeutic options, multiple basic and translational options merit investigation. Our data strengthen the body of evidence for targeting ZIKV replication with nucleotide analog RNA-polymerase inhibitors in general, and SOF in particular. These promising data warrant further preclinical studies of SOF to insure efficacy and safety for congenital ZIKV infection.

## Materials and Methods

### Animals

#### Nod.Scid

8-week-old NOD/SCID (NOD.CB17-Prkdc < scid > /J) mice were purchased from the Jackson Laboratory. Mice received a single intra-venous (IV) injection of 100 μl ZIKV-infected (ibH 30656) Vero cell supernatant containing 10^8^ PFU on day zero (n ≥ 6 mice). The mouse groups were randomized and experimenters were blinded to group assignment and treatment. The control group received a single IV injection of 100 μl of filtered conditioned media from uninfected Vero cells, used as mock-infection (n = 6 mice). Of note, no signs of inflammation that could be attributable to any floating cells captured in the conditioned medium was observed in mock-infected mouse group. The mock-infected animals failed to display any observable side effects from the mock-infection including changes in weight, gross locomotor activity or fur consistency. The next day, infected mice received an intra-peritoneal (IP) injection or oral gavage (PO) of 50 mg/kg/day of SOF (Acme Biosciences, Palo Alto, CA) (diluted in vehicle: DMSO and Tween 80 (0.5% v/v)/polyethylene glycol-400(49.5% v/v) for IP and PO respectively) (n = 3), and the placebo group received only vehicle (n = 3). Although the animal dosages (50 mg/kg) used experimentally exceed the human dose (400 mg) by weight, species scaling by body surface area corresponds to a scaled human equivalent dose (HED) of 4 mg/kg, less than the human SOF dose for a 70 kg individual^36^. Mice were injected with SOF and monitored daily for 10 days. On day 11, mice were euthanized and blood was collected for downstream applications. All animal procedures were approved by the Institutional Animal Care and Use Committee (IACUC) of the University of California. All procedures followed institutional guidelines. For survival experiments, end stage was defined when the mouse had lost 20% of their initial body weight and therefore needed euthanasia.

### Infection of SJL pregnant dams

Timed pregnant (E12.5) SJL mice 2–3 month of age (Jackson Laboratories, Bar Harbor, ME, USA) were infected intravenously (retro-orbital injection, RO) with 200 μl of ZIKV (strain PA 259459) containing 2 × 10^5^ PFU of virus particles as previously described^38^. This dose and timing of the infection during pregnancy was sufficient to cause a robust ZIKV infection in SJL mice^38^. SOF (50 mg/kg) was delivered daily by oral gavage for six days (E13.5–18.5). The animals were euthanized to obtain fetal tissues and collect blood samples from mothers. All the experiments were performed with the approval of the Sanford Burnham Prebys Medical Discovery Institute IACUC Committee protocol AUF#16–049.

### ZIKV NS1 ELISA

A monoclonal antibody-based enzyme-linked immunosorbent assay (ELISA) was performed for the quantitative detection of the Zika Virus nonstructural protein NS1 (BioFront Technologies). Briefly, cell culture supernatants from ZIKV strains ibH 30656, and PA 259459 were collected, lysed, clarified and further incubated in a ZIKV NS1 ELISA assay plate. Anti-NS1 HRP-conjugate and HRP substrate were used as detection method. After adding the quenching solution, absorbance values were acquired (450 nm) and analyzed using a standard curve generated from recombinant Zika Virus (rZIKV) NS1 protein.

### Real-time PCR from the NOD/SCID mice samples

Following euthanasia, whole blood of each mouse was collected and allowed to coagulate at RT for 30 minutes. After coagulation, the blood was centrifuged at 16,000 × *g* for 10 minutes for separation of the serum (~150 μl of serum per mouse). The serum volume was divided into two tubes: (1) to treat the Vero cells for plaque assay and (2) for RNA extraction. To extract the RNA from the serum, QIAamp viral RNA isolation kit (Qiagen) was used according to the manufacturer’s instructions. A total of 250 ng of total RNA was reverse transcribed using QuantiTect Reverse Transcription Kit (Qiagen) including a step of genomic DNA wipe out. Approximately 15 ng of cDNAs were used per reaction and PCRs were carried out in a final volume of 20 μl. Triplicate samples were analyzed in a CFX96 Touch Real-Time PCR Detection System (Bio-rad) using iQ™ SYBR® Green Supermix (Bio-rad). The run method was as follows: 3 minutes at 95 °C, 42 cycles of 10 seconds at 95 °C followed by 30 seconds at 58 °C, and a melting curve was performed to confirm the identity of the amplified product. The primers used are ZIKVF9027: CCT TGG ATT CTT GAA CGA GGA; ZIKVR9197c: AGA GCT TCA TTC TCC AGA TCAA, as previously reported^54^. In all cases, the standard curve method was used for quantitative analysis of expression levels, based on serial dilutions of PCR product amplification of the ZIKV genome.

### Real-time PCR from the SJL mice samples

100 μl of blood samples were obtained from the pregnant (E18) SJL mice. The RNA was extracted from each sample using the NucleoSpin RNA Kit (Macherey-Nagel GmbH, Duren, Germany) accordingly to the manufacturer’s instructions. Purified RNA samples were quantified using NanoDrop spectrophotometer (NanoDrop Technologies, Wilmington, DE, USA) and stored at −80 °C. All real-time assays were performed by using the QuantiTect Reverse Transcription Kit (QIAGEN, Valencia, CA, USA) with amplification in the LightCycler 480 II instrument (Roche, Indianapolis, IN, USA) following the manufacturer’s protocol.

The real-time reaction was performed in triplicates with the LightCycler 480 SYBR Green I Master Mix reagents (Roche, Indianapolis, IN, USA). Primers (ZIKV-835 5′-TTG GTC ATG ATA CTG CTG ATT GC-3′ and ZIKV-911c 5′-CCT TCC ACA AAG TCC CTA TTG C-3′) specific for ZIKV were synthesized by IDT Inc. (San Jose, CA, USA) and were previously described^55^. The amplification was done by: initiation 95 °C for 10 minutes followed by 50 amplification cycles of 95 °C for 15 seconds, 60 °C for 30 seconds, and 72 °C for 30 seconds.

ZIKV RNA from fetuses’ heads were detected using TaqMan® Universal Master Mix II, no UNG (Thermofisher). The primers used are: ZIKV835: TTG GTC ATG ATA CTG CTG ATT GC; ZIKV911c: CCT TCC ACA AAG TCC CTA TTG C and the probe ZIKV860FAM: CGG CAT ACA GCA TCA GGT GCA TAG GAG. The samples were carried out in 20 μl reactions and the run method was as follows: 10 minutes at 95 °C, 42 cycles of 15 seconds at 95 °C followed by 1 minute at 58 °C. The sensitivity of the ZIKV real-time assays was evaluated by titration of serial dilution of virus with previously known titer. Samples with C_t_ values above 40 were excluded from the analysis and represented as non-detected samples (n.d.). GraphPad Prism was used as fitting software.

### Human antiviral response qPCR array analysis in NPCs

500 ng of total RNA from mock, ZIKV (strain ibH 30656)-infected at an MOI of 0.1 and ZIKV-infected treated with 50 μM SOF NPCs were extracted at 120 hours p.i. and submitted to gene expression analysis Human antiviral response genes using the RT2 Profiler PCR Array (cat. no. PAHS-122ZD - Qiagen) according to the manufacturer’s protocols. qPCR was on a CFX96 Touch Real-Time PCR Detection System (Bio-rad). To evaluate gene expression, we established a twofold up- or downregulation compared to mock infected samples. Data was analyzed using the RT^2^ profiler RT–PCR array data analysis software v3.5.

### Viral culture and amplification

The ZIKV strain ibH 30656, isolated from human blood in Nigeria, the strain (PA 259459), isolated from human blood in Panama and Brazil-ZKV2015 isolated from a febrile case in the state of Paraiba (Brazil) were amplified using the African green monkey kidney cells (Vero) and titers determined by plaque assay as previously reported^21^. Both strains of ZIKV isolated from Nigeria and Panama were obtained from the World Reference Center for Emerging Viruses and Arboviruses at the University of Texas Medical Branch, Galveston. Vero cells were cultured in DMEM supplemented with 10% fetal bovine serum (FBS; HyClone, Logan, Utah) and 1% penicillin/streptomycin (P/S). The cells were maintained at 37 °C and 5% of CO_2_.

### Plaque forming assay

Monolayers of Vero cells plated on 48-well plates were exposed to different dilutions of the supernatant from yield-reduction assays for 1 hour at 37 °C. Next, cells were washed with PBS and DMEM containing 1% FBS and 0.4% Agarose (Ultrapure Life technologies) (overlay medium) was added to cells. After 7 days at 37 °C, the monolayers were fixed with 4% of paraformaldehyde in PBS and stained with a 0.1% solution of crystal violet in 70% methanol. The virus titers were calculated by scoring the plaque forming units (PFU) using the following formula: number of plaques/(dilution factor × volume).

### Human stem cell culture

For the generation of NPCs, cells were differentiated and maintained as previously described^56–58^. Two control iPSC lines maintained in mTeSR media were switched to N2 [DMEM/F12 media supplemented with 1x N2 NeuroPlex Serum-Free Supplement (Gemini) with the dual SMAD inhibitors 1 μM of dorsomorphin (Tocris) and 10 μM of SB431542 (Stemgent)] daily, for 48 hours. After two days, colonies were scraped off and cultured under agitation (95 rpm) as embryoid bodies for seven days using N2 media with dorsomorphin and SB431542. Media was changed every other day. Embryoid bodies were then plated on Matrigel-coated dishes, and maintained in DMEM/F12 supplemented with 0.5 × of N2 supplement, 0.5 × Gem21 NeuroPlex Serum-Free Supplement (Gemini), 20 ng/mL basic fibroblast growth factor (bFGF, LifeTechnologies) and 1% penicillin/streptomycin (P/S). After 7 days in culture, formed rosettes from the plated EBs were manually selected, gently dissociated with StemPro Accutase (LifeTechnologies) and plated onto 10 μg/mL poly-L-ornithine (Sigma)/5 μg/mL laminin (LifeTechnologies) coated plates. Neuronal progenitor cells (NPCs) were maintained in DMEM/F12 with N2, Gem21, bFGF and P/S. To induce cortical neuron differentiation, FGF was retrieved as previously described^57^. The cortical neurons were allowed to differentiate for 4 weeks before infection with ZIKV. The media was changed every other day. NPCs were expanded as soon as confluent, using StemPro Accutase for 5 minutes at 37 °C, centrifuged and replated with NGF with a 1:3 ratio in poly-L-ornithine/Laminin-coated plates. All the cell lines tested negative for mycoplasma contamination. All experiments were approved and performed in accordance with the Institutional Review Boards (IRB) and Embryonic Stem Cell Research Oversight (ESCRO) guidelines and regulations.

### *In vitro* infection

NPCs and neurons were infected with the ibH 30656 or Brazil-ZKV2015 strain of ZIKV as described before^21^. The viruses were used at MOIs of 0.1 and 1 for TUNEL assays and MOI of 0.1 or 10 for the neurosphere assays. For mock controls, the same volume of supernatant from Vero cells was added to each experiment. For the vehicle of SOF treatment, equivalent volume of DMSO corresponding to the highest SOF dose was added into the culture media (0.1% of DMSO). For neuronal infection, NPCs were previously differentiated for 28 days and then neurons were infected at an MOI = 10. NPCs and neurons were treated immediately after infection and every 24 hours for the duration of the experiment. SOF (Acme Bioscience AB3793) was added to cell culture supernatant at the desired concentration.

### Neurosphere assay

NPCs were plated and infected as described above. After the infection, fresh media containing different concentrations of SOF (10 and 20 μM) or vehicle (0.1% DMSO) was added. The next day, cells were lifted and cultured under gentle agitation (95 rpm) as neurospheres for 24 hours. At 48, 72, 120 and 180 hours p.i., images of neurospheres were captured using EVOS Cell Imaging System (ThermoFisher) and analyzed with ImageJ software. We measured the neurospheres size and viability over time, in the absence of media change to avoid any viral load decrease in the media. The size of neurospheres was measured as the area (number of pixels) calculated using ImageJ software.

### Histology and immunohistochemistry

Mouse embryo at E18.5 were fixed for 48 hours in 4% formaldehyde in PBS, transferred to sucrose, and embedded in paraffin. Then serial sections of 5 µm were prepared, cutting along the sagittal axis of the embryo. Slides were deparaffinized and rehydrated through a series of xylenes and graded ethanol. Antigen retrieval was performed in a pressure cooker at 7.5 psi placing the slides in 0.1 M Tris-HCl buffer (pH 9) for 15 minutes. Slides were rinsed in room temperature distilled water for 6 times, followed by 5 minutes washing in PBS. Endogenous peroxidase activity was then quenched by incubation with 3% hydrogen peroxide in PBS for 30 minutes at room temperature. Slides were in PBS for 5 minutes washed and incubated over-night at 4 °C with the primary antibody (Flavivirus Group Antigen, Millipore, #MAB10216) diluted 1:250 in Dako Antibody Diluent with Background Reducing Components (Agilent, #S3022). Slides were rinse in PBS three times for 5 minutes and then incubated with the secondary antibody polymer HRP conjugated (Abcam, #ab2891, goat anti mouse) for 30 minutes at room temperature, followed by three times washing in PBS and a 3-minute incubation with the DAB complex, prepared as directed by manufacturer (ImmPACT DAB Peroxidase Substrate, Vector Laboratories, #SK-4105). Slides were washed three times in PBS and three times in water, before being counterstained in Mayer’s hematoxylin, dehydrated with a series of graded ethanol and xylenes and finally mounted. Sections were scanned and acquired using Aperio automated system (Leica) and analyzed with ImageScope (Leica).

### Immunofluorescence and imaging analyses

Cells were fixed with 4% paraformaldehyde for 20 minutes at room temperature. Next, samples were permeabilized in 1×-PBS containing 0.1% (v/v) Triton X-100 for 10 minutes. Fixed cells were next incubated with blocking solution [1% fetal bovine serum, (Life Technologies) in 1xPBS]. After 1 hour, the primary antibodies directed against the following were added: Anti-Flavivirus D1–4G2–4–15 (polyclonal mouse, Millipore, 1:250), cleaved caspase-3 (rabbit, Cell Signaling #9661, 1:400), Nestin (rabbit, Abcam ab105389, 1:500) were added (diluted in blocking solution) and samples were incubated overnight at 4 °C. Slides were then washed two times with 1x-PBS, and incubated with the secondary antibody for 30 minutes at 4 °C. Secondary antibodies (all conjugated to Alexa Fluor 488, 555 and 647) were purchased from Life Technologies and used at a 1:1000 dilution. After the 30 minutes incubation, samples were washed twice (1× -PBS), incubated for 5 minutes with and fluorescent nuclear DAPI stain, and mounted with Slow fade gold antifade reagent (Life Technologies). Samples were imaged using an Axio Observer Z1 Microscope with ApoTome (Zeiss). Captured images were analyzed with Zen software from Zeiss. For TUNEL analysis, NPCs were plated, infected after 24 hours, and fixed 96 hours p.i. with 4% paraformaldehyde for 20 minutes. Samples were permeabilized with 0.25% Triton X-100 for 15 minutes and then stained for TUNEL (Click-iT TUNEL assay kit from Life Technologies). Cells were then blocked with 10% fetal bovine serum for 60 minutes and then incubated in primary antibodies (anti-Nestin antibodies purchased from Abcam and anti-flavivirus antibodies purchased from Millipore) overnight at 4 °C and stained with secondary antibodies and DAPI (Life Technologies, 1:5000) diluted in a phosphate buffer saline (PBS) 1x solution for 5 minutes the following day prior to mounting (Life Technologies, ProLong Gold). Images were blindly collected using an Axio Observer Z1 Microscope with ApoTome (Zeiss) and blindly analyzed with ImageJ software.

### Yield-reduction assay

Vero cells were seeded in 384-well plate and ~90% confluence (4000 cells/well) in 50 µl of media (DMEM with 10% FBS) and NPCs were plated at a density of 10,000 cells/well in 384 well plate. A serial dilution was prepared in stock plate starting at 10 mM with 2-fold dilution and 10 dilution points. 250 nL of compound using an Acoustic Transfer System (EDC Biosystems) were transferred from stock plate in triplicate for each concentration. After adding the compound, the plate was incubated for 10 minutes before infection. NPCs were then infected with ZIKV at an MOI of 1 and Vero cells at an MOI of 5 (ibH 30656) and completed the volume of the wells to 50 µl with DMEM/F12 supplemented with 0.5 × of N2 supplement, 0.5 × Gem21 NeuroPlex Serum-Free Supplement (Gemini), 20 ng/mL basic fibroblast growth factor (bFGF, LifeTechnologies) and 1% penicillin/streptomycin (P/S). After 96 hours of incubation the plates were fixed with 4% formaldehyde and stained with 5 µg/ml of DAPI for 1 hour. The plate was read in the Perkin Elmer ImageXpress Micro XL automated microscope and analyzed by a custom MetaXpress software algorithm able to identify and quantify apoptotic nuclei. The software calculated the number of apoptotic nuclei and the total number of nuclei per well. The apoptotic ratio was calculated dividing the number of apoptotic cells by the total number of cells (percentage of apoptosis). The normalized antiviral activity was calculated based on the average apoptosis in the positive control group (not infected wells, 100% antiviral activity) and the average apoptosis in the negative control group (infected and not treated, 0% antiviral efficacy). The dose-response curve was calculated using GraphPad software with the nonlinear regression curve fit, dose-response inhibition with variable slope (four parameters). For Vero cells, after 6 days of incubation, 5 µl of Resazurin 0.2 mg/ml was added (Santa Cruz Biotecnology, sc-206037), incubated for 1 hour at 37 °C and then the plate was read (excitation/emission -560/590) with EnVision 2104 MultiLabel Reader (PerkinElmer). The IC50 was calculated using Prism GraphPad: non-linear curve fitting with variable slope (four parameters) with the following model:

$${\rm{Y}}={\rm{Bottom}}+({\rm{Top}}-{\rm{Bottom}})/(1+{10}^{\wedge }((\mathrm{LogEC50}-{\rm{X}})\ast {\rm{HillSlope}}{\rm{.}}$$

### Sofosbuvir and GS-331007 analysis

GS-331007 was purchased from Cayman Chemical. Extraction and analysis of GS-331007 was performed using a modified protocol of a previously reported method^59^. Extractions were performed by adding 300 μl of ethyl acetate to 50 μl of serum (6:1 v/v). Samples were then vortexed for 60 seconds followed by centrifugation at 2200 × *g* for 5 minutes. The organic layer (top) was collected and dried under a stream of nitrogen. The extract was then dissolved in 50 µl of 1:1 ACN:H_2_0 and 10 μl was analyzed by liquid chromatography mass spectrometry (LCMS) using a Thermo TSQ Quantiva instrument. LC separation was achieved using a Gemini 5U C18 column (Phenomenex). SOF and GS-331007 was resolved via isocratic flow at 0.4 ml/minute for 5 minutes using 50:50 ACN:H_2_O with 0.1% formic acid as solvent. MS analyses were performed using electrospray ionization (ESI) in positive ion mode with the following source parameters: spray voltage of 3.5 kV, ion transfer tube temperature of 325 °C, and vaporizer temperature of 275 °C. MRM was used to detect SOF (m/z 530.2 → 243, CE = 20 V) and GS-331007 (m/z 261.1 → 113, CE = 13 V). Concentrations of SOF and GS-331007 in serum samples were calculated by referring to a prepared calibration curve.

### ZIKV and HCV representative elements selection

According to a recently updated nomenclature, HCV is classified into 7 genotypes, which are subdivided into 67 epidemiologically diverse subtypes^60,61^. To create the phylogenetic tree, we selected representative elements from ZIKV and HCV. HCV is subdivided in 7 genotypes, the following representative virus were used for phylogenetic analysis: genotype 1-M62321, M67463; genotype 2-D00944, AB047639; genotype 3-D17763, D28917; genotype 4-Y11604, FJ462435; genotype 5-Y13184, AF064490; genotype 6-Y12083, AY859526; genotype 7-EF108306. We also selected a set of representative strains from ZIKV, corresponding to the Asian and the African virus lineages^62^. On total, seven representatives from African lineages (accession numbers: KF268948, KF268950, KF268949, LC002520, AY632535, HQ234500, HQ234501) and five representatives from Asian lineages (accession numbers: KJ776791, KF993678, JN860885, EU545988, HQ234499).

### ZIKV NS5 structural modeling

Residues 396–731 of ZIKV polymerase NS5 (unpublished; PDB ID 5TFR) were structurally aligned with residues 84–386 of HCV NS5 (PDB ID 4WTG; 12) in PyMOL. The rotamer for ZIKV NS5 residue D540 was manually adjusted to match that of HCV; the difference likely arose due to the lack of substrate in the ZIKV NS5 structure. Structures overlays and movies were generated in PyMOL.

### Virus amino acid sequences

Virus amino acid sequences corresponding to the RNA polymerase from both flaviviruses (NS5 protein) and hepacivirus (NS5b protein) were downloaded from ViPR web resource^63^ (URL: https://www.viprbrc.org/), and based on completed sequenced molecules. The downloaded flaviviruses sequences and corresponding accessions are: West Nile Virus (genbank: KC601756^64^), Japanese Encephalitis (genbank: JX131374^65^), Dengue Fever (genbank: NC_002640^66^) and ZIKV (genbank: KU497555^67^). The downloaded Hepacivirus amino acid sequence and corresponding accession is: Hepatitis C Virus (genbank: EF424625^68^). As the RNA polymerase (RNApol) from both flaviviruses and hepaciviruses are contained within NS5 protein and NS5b protein respectively, we extracted only the RNApol amino acid sequences for subsequent analysis.

### Phylogenetic tree construction of RNA polymerase

Amino acid sequences corresponding to RNA polymerase from both Flavivirus and Hepacivirus were subjected to phylogenetic tree analysis using the online software resource Phylogeny.fr^69^. The software is based on a built-in pipeline with chaining programs with recognized accuracy as follow: MUSCLE for multiple alignment^70^, Gblocks for alignment refinement^71^, PhyML for tree building^72^, and TreeDyn for tree rendering^73^. All parameters are set up to default, as they are suitable for the investigated virus^69^.

### RNA polymerase amino acid sequence conservation analysis

Amino acid sequences corresponding to RNA polymerase from both Flavivirus and Hepacivirus were subjected to multiple amino acid sequence alignment for DPP domain detection and sequence conservation using Clustall OWS software available through Jalview 2.9.0^74^. After final sequence alignment, DPP triamino acid sequence was located using textual search.

### Statistical analysis

One-way ANOVA tests followed by a Tukey multiple comparison test were used to compare groups and Student’s t-test to compare means of two groups. For statistical analysis of data containing two variables (time and treatment), two-way ANOVA tests followed by a Tukey multiple comparison test was used. Kaplan-Meier Log Rank test was used for survival curves.

## Electronic supplementary material

Supplementary Info File

Supplementary Video 1

Supplementary Table S1

Supplementary Table S2

## References

[1] Campos GS, Bandeira AC, Sardi SI (2015). Zika virus outbreak, Bahia, Brazil. Emerg. Infect. Dis..

[2] Mlakar J (2016). Zika Virus Associated with Microcephaly. N. Engl. J. Med..

[3] van der Linden V (2016). Description of 13 Infants Born During October 2015–January 2016 With Congenital Zika Virus Infection Without Microcephaly at Birth — Brazil. MMWR. Morb. Mortal. Wkly. Rep..

[4] Xu M (2016). Identification of small-molecule inhibitors of Zika virus infection and induced neural cell death via a drug repurposing screen. Nat. Med..

[5] Retallack H (2016). Zika virus cell tropism in the developing human brain and inhibition by azithromycin. Proc. Natl. Acad. Sci. USA.

[6] Barrows NJ (2016). A Screen of FDA-Approved Drugs for Inhibitors of Zika Virus Infection. Cell Host Microbe.

[7] Abbink, P. *et al*. Protective efficacy of multiple vaccine platforms against Zika virus challenge in rhesus monkeys. *Science* (2016). doi:10.1126/science.aah6157 10.1126/science.aah6157 PMC5237380 27492477

[8] Larocca, R. A. *et al*. Vaccine protection against Zika virus from Brazil. *Nature*, 10.1038/nature18952 (2016). 10.1038/nature18952 PMC5003703 27355570

[9] Reefhuis J (2016). Projecting Month of Birth for At-Risk Infants after Zika Virus Disease Outbreaks. Emerg. Infect. Dis..

[10] Sacramento CQ (2017). The clinically approved antiviral drug sofosbuvir inhibits Zika virus replication. Sci. Rep..

[11] Onorati M (2016). Zika Virus Disrupts Phospho-TBK1 Localization and Mitosis in Human Neuroepithelial Stem Cells and Radial Glia. Cell Rep..

[12] Bullard-Feibelman KM (2017). The FDA-approved drug sofosbuvir inhibits Zika virus infection. Antiviral Res..

[13] Ferreira AC (2017). Sofosbuvir protects Zika virus-infected mice from mortality, preventing short- and long-term sequelae. Sci. Rep..

[14] Sulkowski MS (2014). Daclatasvir plus sofosbuvir for previously treated or untreated chronic HCV infection. N. Engl. J. Med..

[15] Gentile I, Maraolo AE, Buonomo AR, Zappulo E, Borgia G (2015). The discovery of sofosbuvir: a revolution for therapy of chronic hepatitis C. Expert Opin. Drug Discov..

[16] Toussaint-Miller KA, Andres J (2015). Treatment Considerations for Unique Patient Populations With HCV Genotype 1 Infection. Ann. Pharmacother..

[17] Elfiky AA, Elshemey WM, Gawad WA, Desoky OS (2013). Molecular modeling comparison of the performance of NS5b polymerase inhibitor (PSI-7977) on prevalent HCV genotypes. Protein J..

[18] Xu D (2014). Fialuridine induces acute liver failure in chimeric TK-NOG mice: a model for detecting hepatic drug toxicity prior to human testing. PLoS Med..

[19] Li C (2016). Zika Virus Disrupts Neural Progenitor Development and Leads to Microcephaly in Mice. Cell Stem Cell.

[20] Souza BSF (2016). Zika virus infection induces mitosis abnormalities and apoptotic cell death of human neural progenitor cells. Sci. Rep..

[21] Cugola, F. R. *et al*. The Brazilian Zika virus strain causes birth defects in experimental models. *Nature* 1–15, 10.1038/nature18296 (2016). 10.1038/nature18296 PMC4902174 27279226

[22] Lindenbach, B. D. & Rice, C. M. In *Fields virology*, *5th Edition* (2007).

[23] Appleby TC (2015). Viral replication. Structural basis for RNA replication by the hepatitis C virus polymerase. Science.

[24] Garcez, P. P. *et al*. Zika virus impairs growth in human neurospheres and brain organoids. *Science* aaf6116 10.1126/science.aaf6116 (2016). 10.1126/science.aaf6116 27064148

[25] Dang, J. *et al*. Zika Virus Depletes Neural Progenitors in Human Cerebral Organoids through Activation of the Innate Immune Receptor TLR3. *Cell Stem Cell*, 1–8, 10.1016/j.stem.2016.04.014 (2016). 10.1016/j.stem.2016.04.014 PMC5116380 27162029

[26] Tang H (2016). Zika Virus Infects Human Cortical Neural Progenitors and Attenuates Their Growth. Cell Stem Cell.

[27] Wu, K.-Y. *et al*. Vertical transmission of Zika virus targeting the radial glial cells affects cortex development of offspring mice. *Cell Res*., 10.1038/cr.2016.58 (2016). 10.1038/cr.2016.58 PMC4897185 27174054

[28] Reznik, S. E. & Ashby, J. C. R. Sofosbuvir: an anti-viral drug with potential efficacy against Zika infection. *Int*. *J*. *Infect*. *Dis*. **55**, In press (2016). 10.1016/j.ijid.2016.12.011 27988410

[29] Mesci, P. *et al*. Modeling neuro-immune interactions during Zika virus infection. *Hum*. *Mol*. *Genet*. 10.1093/hmg/ddx382 (2017). 10.1093/hmg/ddx382 PMC5886060 29048558

[30] Zmurko, J. *et al*. Substrate selectivity of Dengue and Zika virus NS5 polymerase towards 2′-modified nucleotide analogues. *Antiviral Res*. **55**, In press (2016). 10.1016/j.antiviral.2016.12.021 28041959

[31] Kataoka S (1983). Immunologic aspects of the nonobese diabetic (NOD) mouse. Abnormalities of cellular immunity. Diabetes.

[32] Zmurko J (2016). The Viral Polymerase Inhibitor 7-Deaza-2′-C-Methyladenosine Is a Potent Inhibitor of *In Vitro* Zika Virus Replication and Delays Disease Progression in a Robust Mouse Infection Model. PLoS Negl. Trop. Dis..

[33] Lazear HM (2016). A Mouse Model of Zika Virus Pathogenesis Cell Host &amp; Microbe Resource A Mouse Model of Zika Virus Pathogenesis. Cell Host Microbe.

[34] Rossi, S. L. *et al*. Characterization of a Novel Murine Model to Study Zika Virus. *Am*. *J*. *Trop*. *Med*. *Hyg*. **94**, ajtmh.16-0111 (2016). 10.4269/ajtmh.16-0111 PMC4889758 27022155

[35] Aliota MT (2016). Characterization of Lethal Zika Virus Infection in AG129 Mice. PLoS Negl. Trop. Dis..

[36] Nair AB, Jacob S (2016). A simple practice guide for dose conversion between animals and human. J. basic Clin. Pharm..

[37] Gilead Sciences, I. Highlights of prescribing information. United States Food & Drug Administration Available at: https://www.accessdata.fda.gov/drugsatfda_docs/label/2013/204671s000lbl.pdf (2013).

[38] Shiryaev SA (2017). Repurposing of the anti-malaria drug chloroquine for Zika Virus treatment and prophylaxis. Sci. Rep..

[39] Styer LM (2007). Mosquitoes inoculate high doses of West Nile virus as they probe and feed on live hosts. PLoS Pathog..

[40] Barrows NJ (2016). A Screen of FDA-Approved Drugs for Inhibitors of Zika Virus Infection. Cell Host Microbe.

[41] Oliveira DBL (2016). Prolonged Shedding of Zika Virus Associated with Congenital Infection. N. Engl. J. Med..

[42] Ginhoux F (2010). Fate mapping analysis reveals that adult microglia derive from primitive macrophages. Science.

[43] Mandelbrot L (2015). No perinatal HIV-1 transmission from women with effective antiretroviral therapy starting before conception. Clin. Infect. Dis..

[44] Pasternak B, Hviid A (2010). Use of acyclovir, valacyclovir, and famciclovir in the first trimester of pregnancy and the risk of birth defects. JAMA.

[45] Ellis, C. Center for drug evaluation and research 204671Orig1s000. *Pharmacology Review(S)* (2013).

[46] Nishiura H (2016). A theoretical estimate of the risk of microcephaly during pregnancy with Zika virus infection. Epidemics.

[47] Bhatia HK, Singh H, Grewal N, Natt NK (2014). Sofosbuvir: A novel treatment option for chronic hepatitis Cinfection. J. Pharmacol. Pharmacother..

[48] Broutet N (2016). Zika Virus as a Cause of Neurologic Disorders. N. Engl. J. Med..

[49] Carteaux G (2016). Zika Virus Associated with Meningoencephalitis. N. Engl. J. Med..

[50] Furtado JM, Espósito DL, Klein TM, Teixeira-Pinto T, da Fonseca BA (2016). Uveitis Associated with Zika Virus Infection. N. Engl. J. Med..

[51] Mécharles S (2016). Acute myelitis due to Zika virus infection. Lancet (London, England).

[52] Eyer, L. *et al*. Nucleoside inhibitors of Zika virus. *J*. *Infect*. *Dis*. (2016). doi:10.1093/infdis/jiw226 10.1093/infdis/jiw226 27234417

[53] Potisopon S, Ferron F, Fattorini V, Selisko B (2016). & Canard, B. Substrate selectivity of Dengue and Zika virus NS5 polymerase towards 2′-modified nucleotide analogues. Antiviral Res..

[54] Balm MND (2012). A diagnostic polymerase chain reaction assay for Zika virus. J. Med. Virol..

[55] Lanciotti RS (2008). Genetic and serologic properties of Zika virus associated with an epidemic, Yap State, Micronesia, 2007. Emerg. Infect. Dis..

[56] Chailangkarn, T. *et al*. A human neurodevelopmental model for Williams syndrome. *Nature*, 10.1038/nature19067 (2016). 10.1038/nature19067 PMC4995142 27509850

[57] Marchetto MCN (2010). A model for neural development and treatment of Rett syndrome using human induced pluripotent stem cells. Cell.

[58] Marchetto, M. C. *et al*. Altered proliferation and networks in neural cells derived from idiopathic autistic individuals. *Mol*. *Psychiatry*, 10.1038/mp.2016.95 (2016). 10.1038/mp.2016.95 PMC5215991 27378147

[59] Rezk MR, Basalious EB, Karim IA (2015). Development of a sensitive UPLC-ESI-MS/MS method for quantification of sofosbuvir and its metabolite, GS-331007, in human plasma: Application to a bioequivalence study. J. Pharm. Biomed. Anal..

[60] Jackowiak P (2014). Phylogeny and molecular evolution of the hepatitis C virus. Infect. Genet. Evol..

[61] Smith DB (2014). Expanded classification of hepatitis C virus into 7 genotypes and 67 subtypes: updated criteria and genotype assignment web resource. Hepatology.

[62] Enfissi A, Codrington J, Roosblad J, Kazanji M, Rousset D (2016). Zika virus genome from the Americas. Lancet (London, England).

[63] Pickett BE (2012). ViPR: an open bioinformatics database and analysis resource for virology research. Nucleic Acids Res..

[64] Balakrishnan A, Butte DK, Jadhav SM (2013). Complete Genome Sequence of West Nile Virus Isolated from Alappuzha District, Kerala, India. Genome Announc..

[65] Singha H (2013). Complete genome sequence analysis of Japanese encephalitis virus isolated from a horse in India. Arch. Virol..

[66] Naveca FG (2012). Complete Genome Sequence of a Dengue Virus Serotype 4 Strain Isolated in Roraima, Brazil. J. Virol..

[67] Calvet, G. *et al*. Case Report of detection of Zika virus genome in amniotic fluid of affected fetuses: association with microcephaly outbreak in Brazil. *Lancet Infect*. *Dis*. **3099**, In press (2016). 10.1016/S1473-3099(16)00095-5 26897108

[68] Lu L (2007). Complete genomes of hepatitis C virus (HCV) subtypes 6c, 6l, 6o, 6p and 6q: completion of a full panel of genomes for HCV genotype 6. J. Gen. Virol..

[69] Dereeper A (2008). Phylogeny.fr: robust phylogenetic analysis for the non-specialist. Nucleic Acids Res..

[70] Edgar RC (2004). MUSCLE: multiple sequence alignment with high accuracy and high throughput. Nucleic Acids Res..

[71] Castresana J (2000). Selection of Conserved Blocks from Multiple Alignments for Their Use in Phylogenetic Analysis. Mol. Biol. Evol.

[72] Guindon S, Gascuel O (2003). A simple, fast, and accurate algorithm to estimate large phylogenies by maximum likelihood. Syst. Biol..

[73] Chevenet F, Brun C, Bañuls A-L, Jacq B, Christen R (2006). TreeDyn: towards dynamic graphics and annotations for analyses of trees. BMC Bioinformatics.

[74] Waterhouse AM, Procter JB, Martin DMA, Clamp M, Barton GJ (2009). Jalview Version 2–a multiple sequence alignment editor and analysis workbench. Bioinformatics.

