# Author Correction: Blocking Zika virus vertical transmission

**DOI:** 10.1038/s41598-018-26959-4

**Published:** 2018-06-05

**Authors:** Pinar Mesci, Angela Macia, Spencer M. Moore, Sergey A. Shiryaev, Antonella Pinto, Chun-Teng Huang, Leon Tejwani, Isabella R. Fernandes, Nicole A. Suarez, Matthew J. Kolar, Sandro Montefusco, Scott C. Rosenberg, Roberto H. Herai, Fernanda R. Cugola, Fabiele B. Russo, Nicholas Sheets, Alan Saghatelian, Sujan Shresta, Jeremiah D. Momper, Jair L. Siqueira-Neto, Kevin D. Corbett, Patricia C. B. Beltrão-Braga, Alexey V. Terskikh, Alysson R. Muotri

**Affiliations:** 1 University of California San Diego, School of Medicine, Department of Pediatrics/Rady Children’s Hospital San Diego, Department of Cellular & Molecular Medicine, Stem Cell Program, La Jolla, CA 92037–0695 USA; 2 Sanford Burnham Prebys Medical Discovery Institute, 10901N. Torrey Pines Rd., La Jolla, CA 92037 USA; 3 Salk Institute for Biological Studies, Clayton Foundation Laboratories for Peptide Biology, Helmsley Center for Genomic Medicine, La Jolla, California, USA; 4 University of California San Diego, Skaggs School of Pharmacy and Pharmaceutical Sciences, Center for Discovery and Innovation in Parasitic Diseases, 9500 Gilman Dr., La Jolla, CA 92093, MC 0755 USA; 5 Ludwig Institute for Cancer Research, San Diego Branch, 9500 Gilman Dr., La Jolla, CA 92093, MC 2385 USA; 6 University of California San Diego, Department of Cellular and Molecular Medicine, 9500 Gilman Dr., La Jolla, CA 92093, MC 2385 USA; 7 Graduate Program in Health Sciences, School of Medicine, Pontifícia Universidade Católica do Paraná (PUCPR), Curitiba, Paraná, Brazil; 8 University of São Paulo, Institute of Biomedical Science, Department of Microbiology, Laboratory of Stem Cell and Disease Modeling, São Paulo, SP 05508–000 Brazil; 9 University of São Paulo, School of Arts Sciences and Humanities, Department of Obstetrics, São Paulo, SP 03828–000 Brazil; 10 University of São Paulo, School of Medicine, Center for Cellular and Molecular Therapy (NETCEM), São Paulo, SP 01246–903 Brazil; 11 Division of Inflammation Biology, La Jolla Institute for Allergy & Immunology, La Jolla, CA 92037 USA; 12 Skaggs School of Pharmacy and Pharmaceutical Sciences, University of California, San Diego, La Jolla, CA 92093 USA

Correction to: *Scientific Reports* 10.1038/s41598-018-19526-4, published online 19 January 2018

In this Article, the authors neglected to include some potential conflicts of interest. The Competing Interests section should read:

“Dr. Muotri is a co-founder and has equity interest in TISMOO, a company dedicated to genetic analysis focusing on therapeutic applications customized for the disorder autism spectrum and other neurological disorders origin genetics. The terms of this arrangement have been reviewed and approved by the University of California, San Diego in accordance with its conflict of interest policies.”

