# Supplementary material for: Blocking Zika virus vertical transmission
Source: Sci Rep. 2018 Jan 19;8:1218. doi: 10.1038/s41598-018-19526-4 (PMC5775359; doi:10.1038/s41598-018-19526-4)
Supplement: Supplementary file 1 — Supplementary Info File [file 41598_2018_19526_MOESM1_ESM.pdf]

Supplementary Information

Blocking Zika virus vertical transmission

Pinar Mesci[1*], Angela Macia[1*], Spencer M. Moore[1*], Sergey A. Shiryaev[2], Antonella Pinto[2], Chun-Teng Huang[2], Leon Tejwani[1], Isabella R. Fernandes[1], Nicole A. Suarez[1], Matthew J. Kolar[3], Sandro Montefusco[4], Scott C. Rosenberg[5,6], Roberto H. Herai[7], Fernanda R. Cugola[8,9,10], Fabiele B. Russo[8,9,10], Nicholas Sheets[11], Alan Saghatelian[3], Sujan Shresta[11], Jeremiah D. Momper[12], Jair L. Siqueira-Neto[4], Kevin D. Corbett[5], Patricia C. B. Beltrão-Braga[8,9,10¶], Alexey V. Terskikh[2¶], & Alysson R. Muotri[1¶]

[1] University of California San Diego, School of Medicine, Department of Pediatrics/Rady Children's Hospital San Diego, Department of Cellular & Molecular Medicine, Stem Cell Program, La Jolla, CA 92037-0695, USA.

[2] Sanford Burnham Prebys Medical Discovery Institute, 10901 N. Torrey Pines Rd., La Jolla, CA 92037.

[3] Salk Institute for Biological Studies, Clayton Foundation Laboratories for Peptide Biology, Helmsley Center for Genomic Medicine, La Jolla, California, USA.

[4] University of California San Diego, Skaggs School of Pharmacy and Pharmaceutical Sciences, Center for Discovery and Innovation in Parasitic Diseases, 9500 Gilman Dr., La Jolla, CA 92093, MC 0755, USA.

[5] Ludwig Institute for Cancer Research, San Diego Branch, 9500 Gilman Dr., La Jolla, CA 92093, MC 2385, USA.

[6] University of California San Diego, Department of Cellular and Molecular Medicine, 9500 Gilman Dr., La Jolla, CA 92093, MC 2385, USA.

[7] Graduate Program in Health Sciences, School of Medicine, Pontifícia Universidade Católica do Paraná (PUCPR), Curitiba, Paraná, Brazil.

[8] University of São Paulo, Institute of Biomedical Science, Department of Microbiology, Laboratory of Stem Cell and Disease Modeling, São Paulo, SP, 05508-000, Brazil.

[9] University of São Paulo, School of Arts Sciences and Humanities, Department of Obstetrics, São Paulo, SP, 03828-000, Brazil.

[10] University of São Paulo, School of Medicine, Center for Cellular and Molecular Therapy (NETCEM), São Paulo, SP, 01246-903, Brazil.

[11] Division of Inflammation Biology, La Jolla Institute for Allergy & Immunology, La Jolla, CA 92037, USA.

[12] Skaggs School of Pharmacy and Pharmaceutical Sciences, University of California, San Diego, La Jolla, CA, 92093.
*These authors contributed equally to this work.
¶Lead Contact: Dr. Alysson R. Muotri, 2880 Torrey Pines Scenic Drive, La Jolla, CA 92037-0695, Email: muotri@ucsd.edu, Phone: (858) 534-9320.

Keywords: Zika virus, NS5B inhibitor, Sofosbuvir, antiviral treatment, nucleoside/nucleotide analog, vertical transmission.

Running title: Sofosbuvir blocks Zika virus vertical transmission

Supplementary Figures

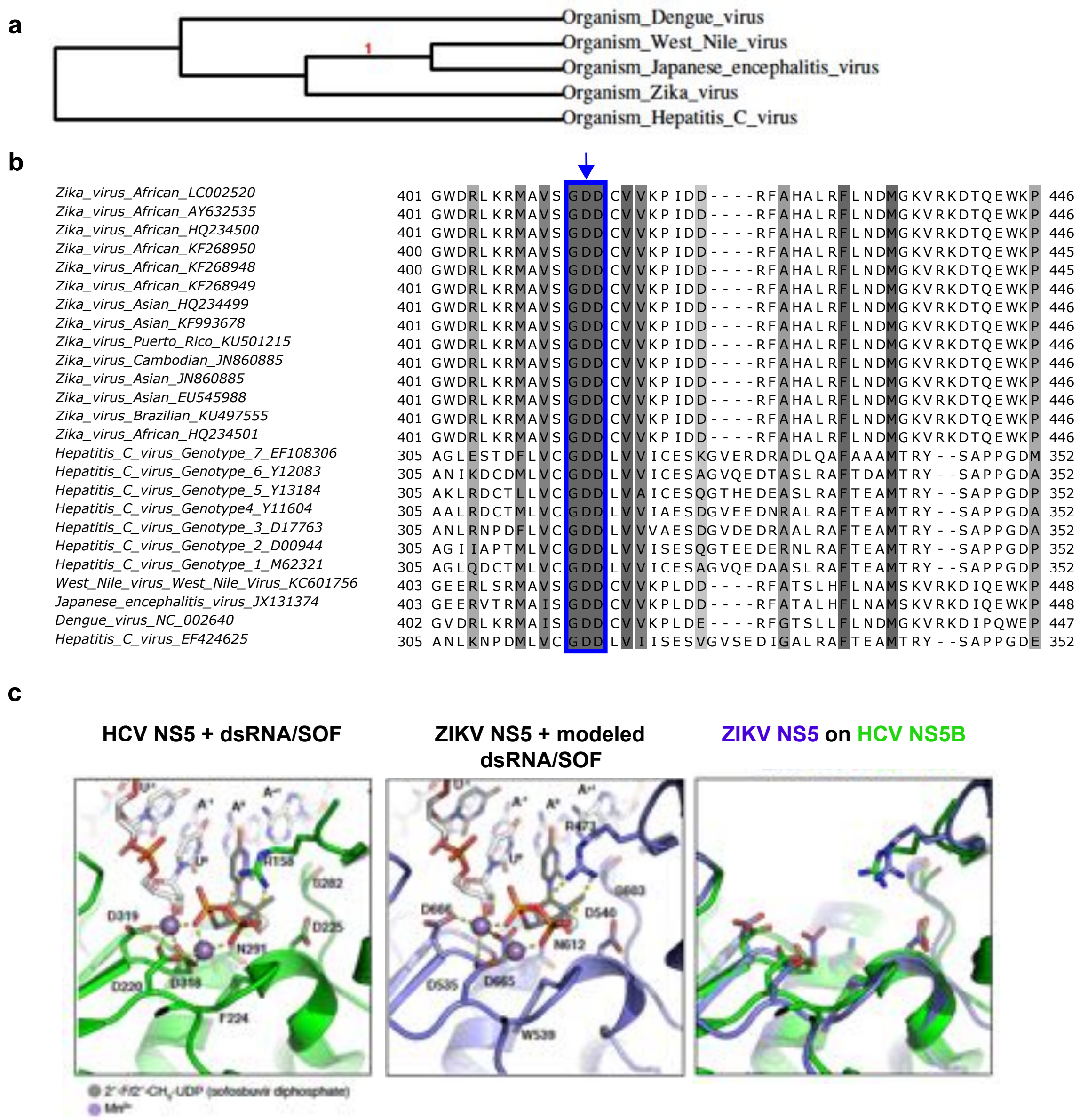

Supplementary Figure 1 | The RdRp domain is similar between different members of *Flaviviridae* family. A. A cladogram tree based on amino acid sequence of the NS5 polymerase region of several members of the flavivirus and hepacivirus genus including Dengue, West Nile, Japanese encephalitis and ZIKV for flavivirus and Hepatitis C for

hepacivirus. **B.** Amino acid sequences of polymerase from ZIKV and other viruses. Amino acid sequences correspond to polymerase-related peptides of NS5 region for flavivirus or NS5b for Hepacivirus. **C.** Close-up of the HCV NS5 polymerase active site (green) in complex with dsRNA, SOF diphosphate (gray with 2' fluorine atom shown as light green sphere), and Mn2+ ions (purple spheres) (PDB ID 4WTG). Activesite residues, including D318/319 of the GDD motif, are shown as sticks (left panel). Close-up of the ZIKV NS5 polymerase active site, with modeled dsRNA, SOF diphosphate (gray with 2' fluorine atom shown as light green sphere), and Mn2+ ions (purple spheres) based on superposition with the structure of HCV NS5 bound to these ligands (PDB ID 4WTG). The only change necessary to accommodate these ligands in ZIKV NS5 is a manual adjustment of the D540 rotamer (middle panel). HCV and ZIKV NS5 overlaid, with dsRNA and SOF removed for clarity (right panel).

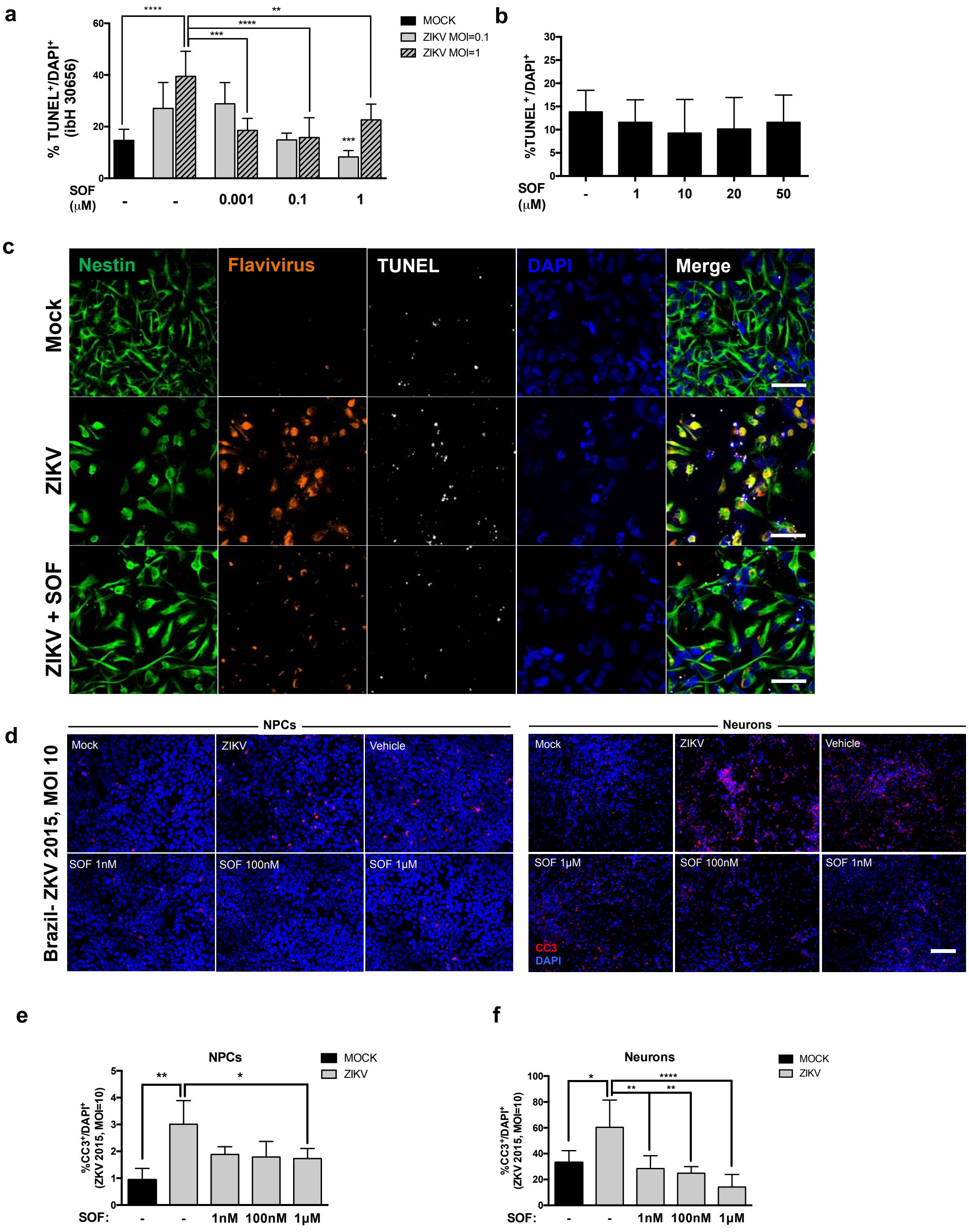

Supplementary Figure 2 | SOF treatment rescues apoptotic NPCs and neurons. A.

Percentages of apoptotic cells were calculated after 96 hours p.i. (MOI = 0.1 and MOI =

1), averaged, and graphed accordingly in the presence of SOF at different doses (1 nM, 100 nM and 1 µM). One-way ANOVA tests with Tukey multiple comparison were performed to compare different groups. The presented values are means of $TUNEL^+/DAPI^+$ percentage ± SD (n = 6 images captured per condition), $^{**}P < 0.01$, $^{***}P < 0.001$ and $^{****}P < 0.0001$. **B**. The percentages of apoptotic cells were calculated in mock-infected cells, averaged, and graphed accordingly in the presence of vehicle or SOF at 1, 10, 20 and 50 µM. Note that the vehicle (-) and SOF treatments did not have any effect on apoptosis of NPCs. One-way ANOVA tests with Tukey multiple comparison were performed to compare with vehicle group. The presented values are means of $TUNEL^+/DAPI^+$ percentage ± SD (n = 6 images captured per condition). **C**. Representative images of immunostainings of NPCs (MOCK, ZIKV-infected, ZIKV-infected and SOF treated). Note that the flavivirus staining cross-reacting with ZIKV and the TUNEL stainings are decreasing upon SOF treatment. Scale bar 20 µm. **D.** Representative images of Cleaved-Caspase 3 (CC3), an early apoptotic cell marker, and DAPI immunostainings of NPCs (left panel) and 4 week-old cortical neurons (right panel) infected with ZIKV (Brazil-ZKV 2015, MOI = 10). Note that CC3 staining decreases upon treatment with SOF. Scale bar 50 µm. **E-F.** The percentages of apoptotic cells were calculated after 96 hours p.i. ZIKV infection (MOI = 10) in NPCs (**E**) and in neurons (**F**) by averaging the integrated intensity of CC3 staining, and were graphed accordingly in the presence of SOF at different doses (1 nM, 100 nM and 1 µM). One-way ANOVA tests with Tukey multiple comparisons were performed to compare with vehicle-treated ZIKV-infected group. The presented values are means of $CC3^+$ over the total amount of DAPI percentage ± SD (n = 6 images captured per condition), $^{*}P < 0.05$, $^{**}P < 0.01$, $^{***}P < 0.001$, $^{****}P < 0.0001$.

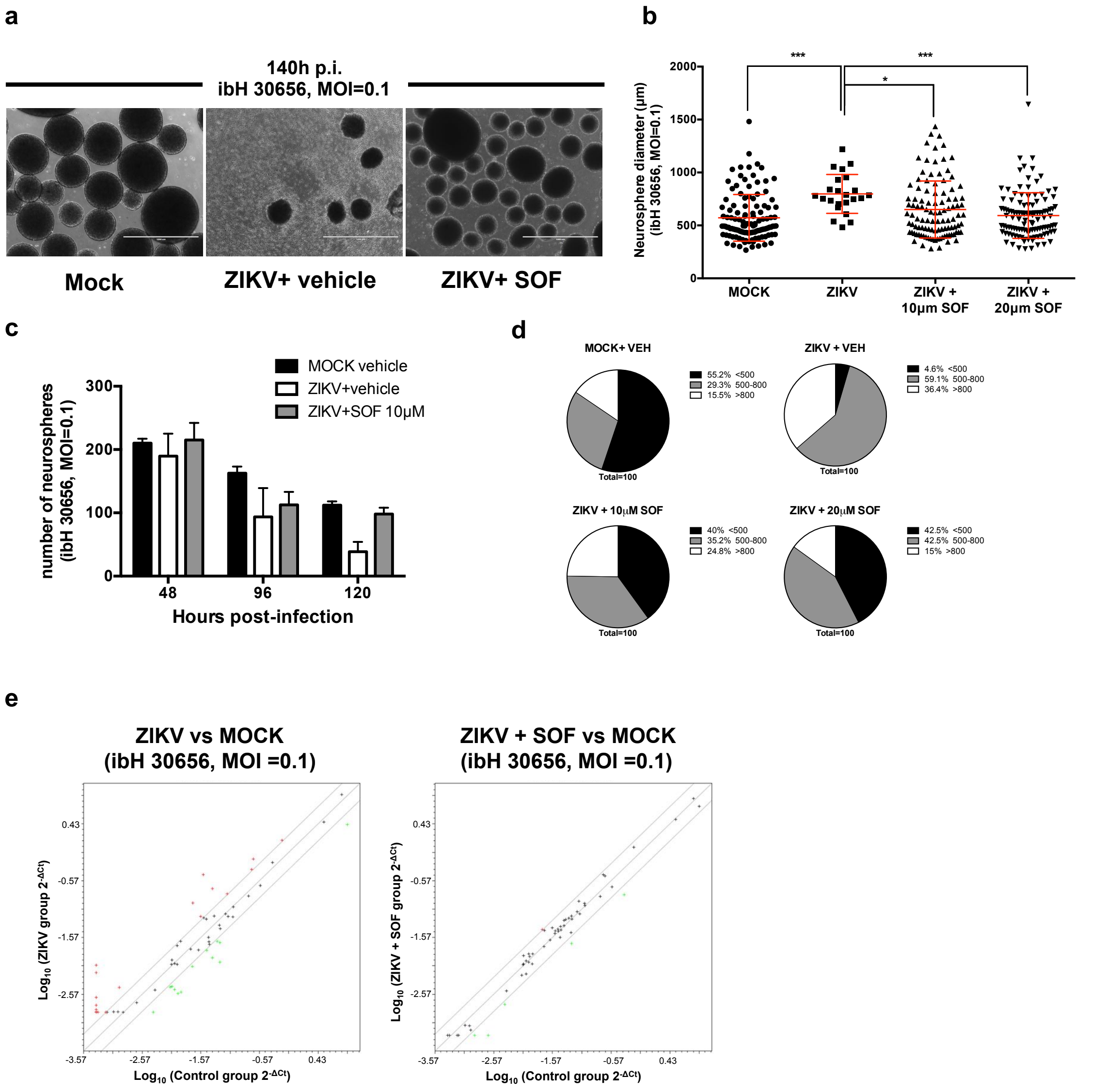

Supplementary Figure 3 | Impact of SOF on human neurospheres. A.

Representative images of mock-infected human neurospheres (left panel), ZIKV-infected vehicle-treated neurospheres (middle panel) and ZIKV-infected SOF-treated (20 µM) (right panel) neurospheres image (ibH30656, MOI = 0.1, 140 hours p.i.), scale bar: 500 µm. **B**. Quantification of neurosphere diameter treated with vehicle or different doses of SOF (10 µM and 20 µM), 140 hours p.i. One-way ANOVA tests with Tukey multiple comparison were performed to compare different groups. The dots represent the number

of spheres counted averaged and plotted for each condition ± SD (n = 5 images captured per condition), **P* < 0.05, ****P* < 0.001 (n ≥ 100 neurospheres counted per condition). **C**. Neurosphere amount was counted over time after infection with ZIKV (ibH30656, MOI = 0.1) (48, 96 and 120 hours p.i.) treated with vehicle or with SOF (10 µM). Bars represent the number of neurospheres ± SD at a given time (48, 96, and120 hours p.i.). Two-way ANOVA tests with Tukey multiple comparison were performed to compare different groups. **D**. The size distribution of mock-infected neurospheres, ZIKV-infected vehicle (VEH)-treated, ZIKV-infected and SOF treated (10 µM and 20 µM) at 140 hours p.i. **E**. Scatter plot of human antiviral response genes in ZIKV versus MOCK and ZIKV+SOF (50 µM) versus MOCK infected NPCs (ibh 30656, MOI = 0.1, 120 hours p.i.) threshold = two-fold compared to the MOCK group. Upregulated genes are above the threshold (in red) and downregulated genes are below the threshold (in green) as analyzed by RT^2 Profiler PCR Array Data Analysis version 3.5.

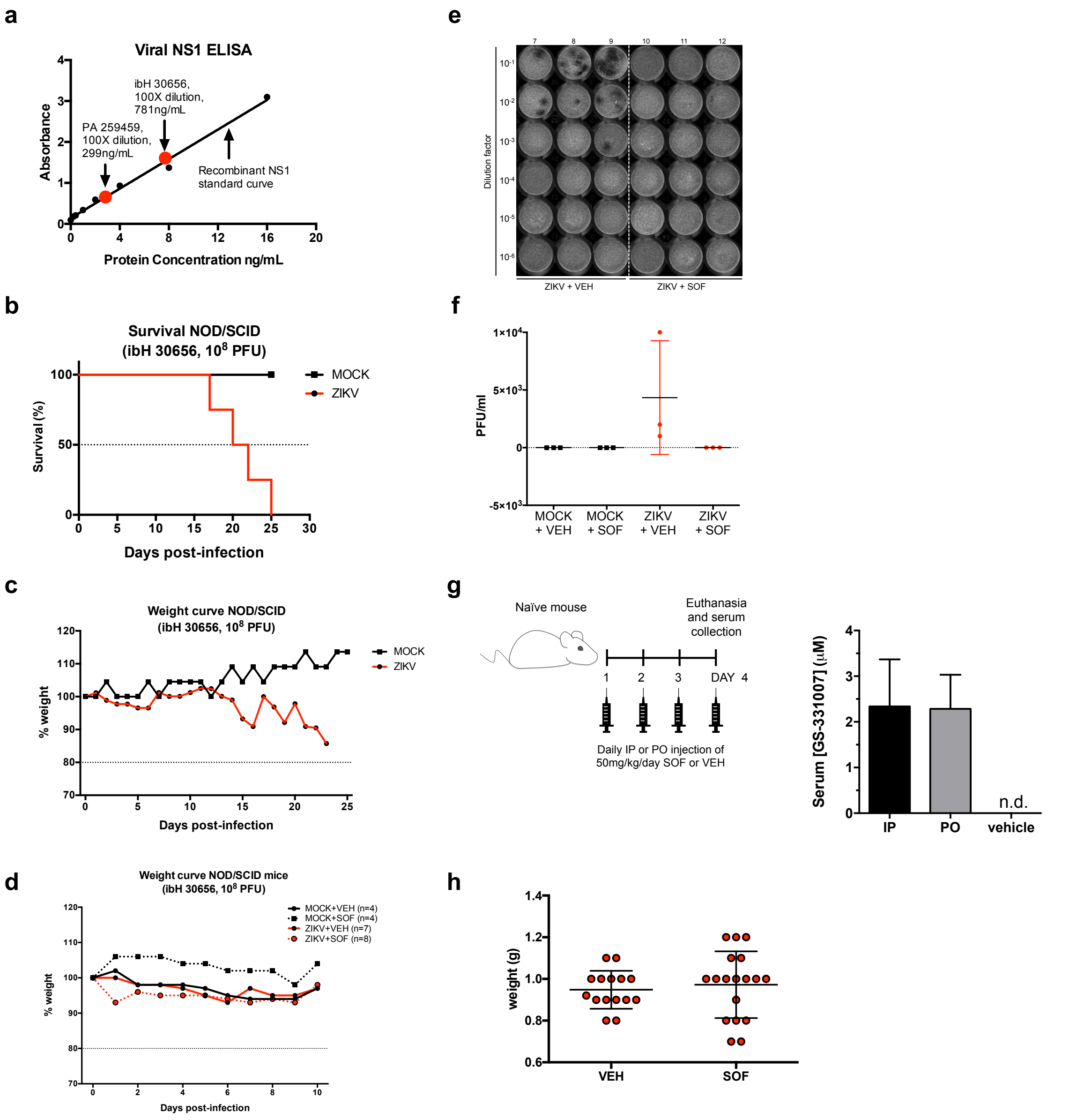

Supplementary Figure 4| SOF treatment in immunodeficient and immunocompetent mice. A. Protein concentration of ZIKV iBH 30656 and PA 259459 strains interpolated as 781ng/mL and 299ng/mL, respectively, from ELISA targeting viral nonstructural protein 1 (NS1). **B.** Kaplan-Meier survival curve of mock and ZIKV-infected NOD/SCID mice (ibH 30656, 108 PFU, IV). The mean age of survival was 21± 3.36 days in ZIKV-infected group while in the mock group, all the mice survived (n = 4 mice per

group) (p=0.0067). **C.** Percentage of weight curve of ZIKV- and MOCK-infected mice (n = 4 mice per group). ZIKV-infected mice started to lose weight on day 11 progressively until end stage. **D.** Percentage of weight of NOD/SCID mice during 10 days after infection. There was no decrease in total body weight in either groups mock + VEH (n = 3 mice), mock + SOF (n = 3 mice), ZIKV + VEH (n = 3 mice) and ZIKV + SOF (n = 3 mice). **E.** Image of the plaque assay performed on NOD/SCID mouse serum 10 days p.i. Note that serum samples of ZIKV, SOF-treated mice do not form any plaques (0/3 mice) while vehicle-treated ones all form plaques (3/3 mice), each column contains serum from one mouse at different serial dilutions. **F.** Quantification of the plaque assay (PFU/ml) performed on NOD/SCID mouse serum 10 days p.i. Each dot represents a mouse (n = 3 mice per group). Error bars are ± SD. **G.** Schematic of experimental design for assessing serum levels of the active metabolite of SOF, GS331007. Naïve mice (n = 4 per group) randomly assigned to receive 50 mg/kg of SOF by IP or by PO for four days and were euthanized on day 4 after the last administration. Serum levels of GS331007 were determined by liquid chromatography-mass spectrometry. Bars represent means ± SD. **H.** No treatment-dependent differences observed in mean weight of offspring of ZIKV-infected SJL animals treated with VEH or SOF (n=15-18 per group, one dot per fetus).

Supplementary Table S1| SOF treatment in ZIKV-infected NPCs (Related to Figure 2). A. Absolute quantification of qRT-PCR-amplified ZIKV-genome in vehicle or SOF-treated infected NPCs (ibH 30656, MOI = 0.1) at different concentrations over time. Standard curves were generated by serial dilutions of a known number of copies of ZIKV-genome. For comparison purposes, viral genome copies were relativized to ng of RNA sample. **B.** Relative quantification of qRT-PCR-amplified human antiviral response genes in MOCK, ZIKV and ZIKV+SOF (50 μM) NPCs (ibh 30656, MOI = 0.1, 120 hours p.i.).

Supplementary Table S2| SOF blocks ZIKV replication in vivo (Related to Figure 3). Absolute quantification of ZIKV RNA viral load measured in the serum and heads of infected mice by qRT-PCR. Standard curves were generated by serial dilutions of a known number of copies of ZIKV-genome. Samples with Ct values above 40 were excluded from the analysis and represented as non-detected samples (n.d.). Amplification curves were analyzed for true amplification. When unusual curves were observed in both linear and exponential scales, samples were assigned as non-detected samples. For comparison purposes, viral genome copies were relativized to ng or ug of RNA sample.

Supplementary Video 1| ZIKV NS5 structural modeling.
Structure of Hepatitis C virus NS5 polymerase (green, Appleby et al. 2015, PDB ID 4WTG) in complex with dsRNA, sofosbuvir diphosphate (gray), and Mn2+ ions (purple spheres). Zika NS5 polymerase (blue, PDB ID 5TFR) overlaid with the HCV polymerase, highlighting the conserved active site residues. Structure of Zika NS5 polymerase (blue) with sofosbuvir modeled into the active site.
